# Barley *Viridis-k* links an evolutionarily conserved C-type ferredoxin to chlorophyll biosynthesis

**DOI:** 10.1093/plcell/koab150

**Published:** 2021-05-29

**Authors:** David Stuart, Malin Sandström, Helmy M. Youssef, Shakhira Zakhrabekova, Poul Erik Jensen, David Bollivar, Mats Hansson

**Affiliations:** 1 Department of Biology, Lund University, Lund 22362, Sweden; 2 Faculty of Agriculture, Cairo University, Giza 12613, Egypt; 3 Department of Food Science, University of Copenhagen, Frederiksberg DK-1958, Denmark; 4 Department of Biology, Illinois Wesleyan University, Bloomington, IL 61702-2900, USA

## Abstract

Ferredoxins are single-electron carrier proteins involved in various cellular reactions. In chloroplasts, the most abundant ferredoxin accepts electrons from photosystem I and shuttles electrons via ferredoxin NADP^+^ oxidoreductase to generate NADPH or directly to ferredoxin dependent enzymes. In addition, plants contain other isoforms of ferredoxins. Two of these, named FdC1 and FdC2 in *Arabidopsis thaliana*, have C-terminal extensions and functions that are poorly understood. Here we identified disruption of the orthologous *FdC2* gene in barley (*Hordeum vulgare* L.) mutants at the *Viridis-k* locus; these mutants are deficient in the aerobic cyclase reaction of chlorophyll biosynthesis. The magnesium-protoporphyrin IX monomethyl ester cyclase is one of the least characterized enzymes of the chlorophyll biosynthetic pathway and its electron donor has long been sought. Agroinfiltrations showed that the *viridis-k* phenotype could be complemented in vivo by *Viridis-k* but not by canonical ferredoxin. VirK could drive the cyclase reaction in vitro and analysis of cyclase mutants showed that in vivo accumulation of VirK is dependent on cyclase enzyme levels. The chlorophyll deficient phenotype of *viridis-k* mutants suggests that VirK plays an essential role in chlorophyll biosynthesis that cannot be replaced by other ferredoxins, thus assigning a specific function to this isoform of C-type ferredoxins.

## Teaser

The aerobic Mg-protoporphyrin IX monomethyl ester cyclase of the chlorophyll biosynthetic pathway obtains electrons from a ferredoxin with a C-terminal extension.

**Figure:**
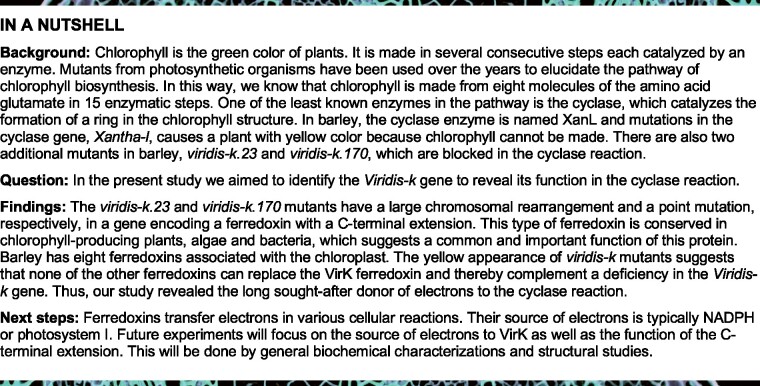


## Introduction

**Figure 1:**
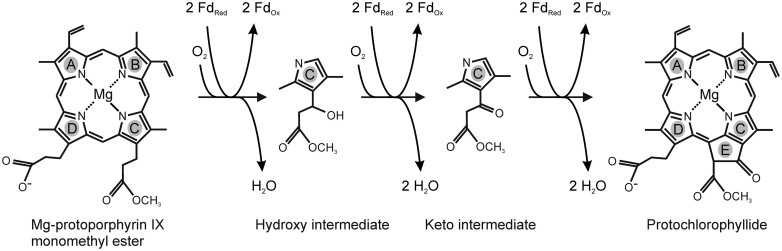
Proposed intermediates in the aerobic MPE cyclase reaction. The reaction has been proposed to proceed via β-hydroxy and β-keto intermediates. The first step is a hydroxylation reaction which bears some resemblance to the reaction catalyzed by soluble methane monooxygenase (Banerjee et al., 2019). Both the cyclase and the soluble methane monooxygenase use electrons from ferredoxin and their substrate (MPE and methane, respectively) to reduce molecular oxygen to water allowing for the oxidations leading to production of the hydroxy intermediate and methanol, respectively. The electrons in the further oxidation of the hydroxy intermediate and the keto intermediate to protochlorophyllide are also used together with additional electrons from ferredoxin to reduce oxygen.

The chlorophyll molecule is magnesium (Mg)-containing tetrapyrrole synthesized in several consecutive steps each catalyzed by a specific enzyme (Tanaka and Tanaka, 2007; Bryant et al., 2020). One of the least understood steps is the conversion of Mg-protoporphyrin IX monomethyl ester (MPE) to protochlorophyllide, which is a six-electron oxidation. In this reaction, the fifth ring E is formed which is unique to chlorophylls. The reaction is catalyzed by an MPE cyclase, adding a carbonyl group to C13^1^ as well as forming a carbon–carbon bond between C13^2^ and the bridge carbon between rings C and D (Figure 1). There are two unrelated enzymes that perform this reaction. One is an anaerobic enzyme where the C13^1^ carbonyl group comes from water and the other is an oxygen-dependent enzyme where the carbonyl group instead comes from molecular oxygen (Walker et al., 1989; Porra et al., 1995; Wiesselmann et al., 2020). It is the latter enzyme, from here on just the cyclase, which is found in plants (Tottey et al., 2003; Chen et al., 2017) and is of special interest to this study. This enzyme is a carboxylate bridged diiron monooxygenase and contains a characteristic iron-binding motif EX_n_EXXHX_n_EX_n_EXXH where two iron atoms are bound by the glutamates and histidines (Berthold and Stenmark, 2003). This type of enzyme requires a reductant and the plant cyclase is reduced with electrons from ferredoxin (Stuart et al., 2020; Figure 1).

Plants and algae contain multiple chloroplast ferredoxins that are iron–sulfur proteins of the Fe_2_S_2_ type and catalyze single electron transfer reactions. The iron–sulfur cluster is coordinated by cysteines in a CX_4_CX_2_CX_n_C motif. The pattern of chloroplast ferredoxin expression, in different parts and under different conditions, suggests that they play important functions in various metabolic pathways since they are central electron donors in chloroplast metabolism (Terauchi et al., 2009; Hanke and Mulo, 2013). The classical ferredoxin participates in photosynthesis and passes electrons to ferredoxin NADP^+^ oxidoreductase (FNR), which in turn reduces NADP^+^ to NADPH (Tagawa and Arnon, 1962). However, ferredoxins can directly donate electrons to ferredoxin dependent enzymes involved in processes such as sulfur and nitrogen assimilation, tetrapyrrole metabolism, and fatty acid biosynthesis to name a few (Hanke and Mulo, 2013). Plants contain four major types of conserved ferredoxins (Hanke et al., 2004; Voss et al., 2011). The leaf-type is primarily expressed in photosynthetic tissues and accepts electrons from photosystem I as a part of photosynthetic electron transport while root-type is expressed mainly in nonphotosynthetic tissues and is tuned for accepting electrons from NADPH via FNR in order to support ferredoxin dependent enzymes (Shinohara et al., 2017). In addition to these canonical ferredoxins, plants contain two ferredoxins with an extended C-terminus and are thus referred to as FdC1 and FdC2 in *Arabidopsis thaliana* (Voss et al., 2011). Their functions are not well established. The FdC1 isoform has been shown to accept electrons from photosystem I or NADPH via FNR but does not seem to be able to reduce NADP^+^ via FNR (Voss et al., 2011; Guan et al., 2018). Similarly, FdC2 can also be reduced by photosystem I or NADPH via FNR and may be found in redox-dependent stromal ribonucleoprotein complexes (Kolton et al., 2011).

**Figure 2:**
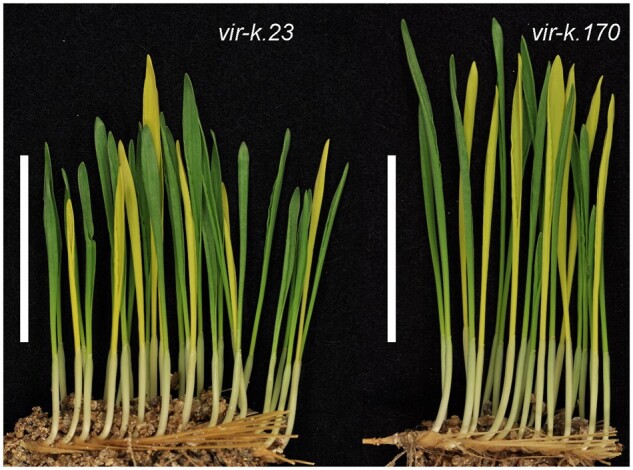
Segregation of *viridis-k* mutants. Ten-day-old seedlings germinated from a spike of heterozygous plants of *vir-k.23* and *vir-k.170*. Mutants homozygous for the recessive lethal *viridis-k* loss-of-function alleles are yellow due to deficiency in chlorophyll biosynthesis. The white bar is 5 cm.

An in vitro cyclase assay utilizing recombinant proteins was recently developed (Stuart et al., 2020). The electrons for the reaction were provided by ferredoxin. In addition, it was found that the recombinant cyclase was only obtained in an active form when coexpressed with *Ycf54* but Ycf54 itself did not seem to be required during catalysis (Stuart et al., 2020). Numerous in vivo studies have also demonstrated the importance of Ycf54 for the cyclase (Hollingshead et al., 2016; Chen et al., 2017, 2018; Chen and Hunter, 2020). In barley (*Hordeum vulgare* L.), the catalytic subunit of the cyclase is the XanL protein encoded by the *Xantha-l* gene. Three barley mutants, *xan-l.35*, *xan-l.81*, and *xan-l.82*, which are defective in the cyclase reaction due to mutations in the *Xantha-l* gene have been described previously (Rzeznicka et al., 2005). In addition, two mutants in the *Viridis-k* gene, *vir-k.23* and *vir-k.170* (Figure 2), have been isolated (Simpson and von Wettstein, 1980) and determined to be deficient in the cyclase reaction (Steccanella et al., 2015). Similar to the *xantha-l* mutations, the *viridis-k* mutations are lethal and have to be maintained in heterozygous stocks. Previous studies have established that the *Viridis-k* gene is clearly different from the *Xantha-l* gene and that it does not code for Ycf54 (Bollivar et al., 2014). We, therefore, set out to identify the *Viridis-k* gene to determine what role this additional component has in the cyclase reaction. As it turns out, the *Viridis-k* gene encodes the barley ortholog of Arabidopsis FdC2.

## Results

### *Viridis-k* is located on chromosome 4H

**Figure 3:**
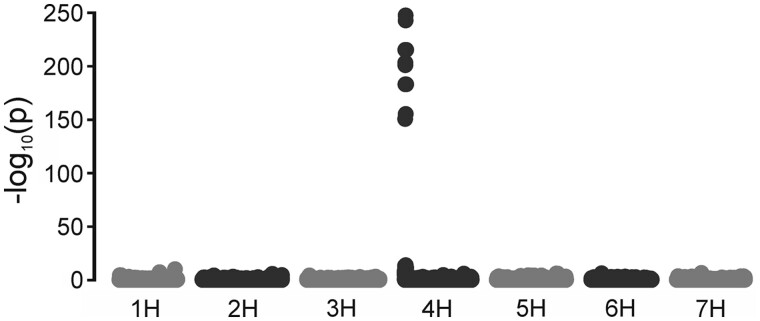
Manhattan plot of genome-wide association scan for chlorophyll deficiency in an F_2_-mapping population. The F_2_ population originated from a cross between mutant *vir-k.23* and barley cultivar Quench. The significance level of SNP-marker trait association (−log_10_ of *P*-values) is shown as a function of the genomic location of the markers on the seven barley chromosomes (1H–7H). The Bonferroni corrected significance threshold is −log_10_(*P* = 0.001/58903) > 7.8. The analysis strongly suggests *Viridis-k* to be in the telomeric region of chromosome 4H.

In order to map the location of the *Viridis-k* gene we constructed an F_2_-mapping population by crossing the mutant *vir-k.23* to the barley cultivar Quench. As nothing was known previously about the location of the *Viridis-k* gene in the barley genome, we used a genotyping-by-sequencing approach on the F_2_-mapping population to obtain single-nucleotide polymorphism (SNP) genotypes distributed throughout the whole genome. We sequenced 30 F_2_ individuals (Supplemental Table S1) and obtained approximately 700 million paired-end reads in total. Raw reads were demultiplexed to assign reads to the correct individuals and to remove reads with errors in the barcode or the restriction enzyme recognition sequence. Overall, roughly 90% of reads were retained after demultiplexing. The analysis of SNPs showed a single region associated with the *viridis-k* phenotype on the short arm of chromosome 4H (Figure 3) which is expected from a trait that is controlled by a single locus. This region was approximately 9.6 Mbp long and located between base pair positions 6.5–16.1 Mbp on chromosome 4H. The region showed complete linkage to the *viridis-k* phenotype (Figure 2) and thus represented the interval where the *Viridis-k* gene is located.

### Identification of a *Viridis-k* candidate gene

In order to narrow down the interval where the *Viridis-k* gene is located a larger F_2_ population of roughly 300 individuals from the *vir-k.23* × Quench cross were genotyped with Cleaved Amplified Polymorphic Sequences (CAPSs) markers (Supplemental Table S2) designed for SNPs discovered during genotyping-by-sequencing. The recombination frequency between each marker and the *Viridis-k* locus was used to calculate the genetic distance measured in centi-Morgans (cM) between the marker and the *vir-k.23* mutation. This mapping was able to narrow down the interval to between 8.7–16.1 Mbp on chromosome 4H representing a region of ∼7.4 Mbp. The two flanking markers located at 8.7 and 16.1 Mbp were calculated to be located at a genetic distance of 6.7 and 2.1 cM away from the *Viridis-k* gene, respectively. Surprisingly, a dramatic drop in recombination rate was observed that resulted in markers at 9.7, 10, and 13.7 Mbp showing a genetic distance of 0 cM to the *Viridis-k* gene (Supplemental Table S3). This region contained 149 high-confidence genes (Supplemental Data Set S1). The best candidate gene in the interval was the ferredoxin orthologous to Arabidopsis *FdC2*. As the cyclase is a ferredoxin-dependent enzyme, there is a direct functional link between FdC2 and the biochemistry of the cyclase reaction (Stuart et al., 2020). We, therefore, investigated if the barley gene orthologous to *FdC2* contained any mutations in the two available *viridis-k* mutants.

### Identification of genetic changes in *viridis-k* mutants

**Figure 4:**
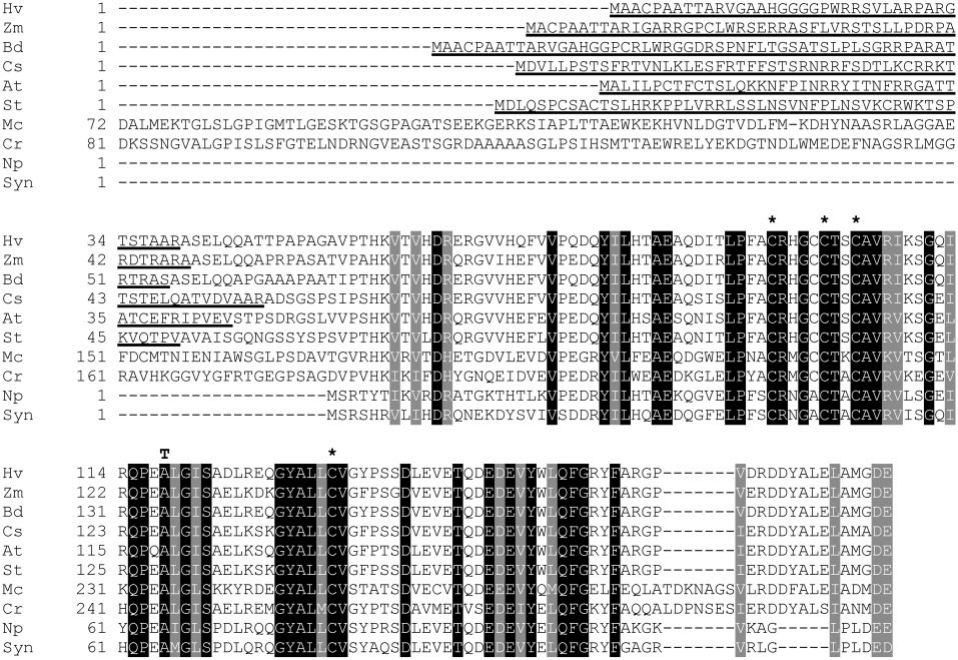
Amino-acid sequence alignment of VirK orthologs. Identical residues and residues with similar physical properties are boxed in black and gray, respectively. Cysteine residues participating in iron–sulfur cluster binding are indicated by asterisks. The Ala-118-Thr substitution caused by the *vir-k.170* mutation is indicated above the barley sequence. Sequences were aligned with Clustal Omega (Sievers et al., 2011). Chloroplast transit peptide sequences (underlined) were predicted by ChloroP (Emanuelsson et al., 1999). Hv, *H. vulgare* KAE8819615; Zm, *Z. mays* ACG28100; Bd, *Brachypodium distachyon* XP_003561243; Cs, *Cucumis sativus* XP_004148487; At, *A. thaliana* NP_0174533; St, *Solanum tuberosum* XP_006352847; Mc, *Micromonas commoda* XP_002501828 (the initial 71 residues have been omitted from the alignment); Cr, *C. reinhardtii* XP_001702961 (the initial 80 residues have been omitted); Np, *Nostoc punctiforme* WP_012407904; Syn, *Synechocystis* sp. PCC 6803 WP_010872870.

**Figure 5:**
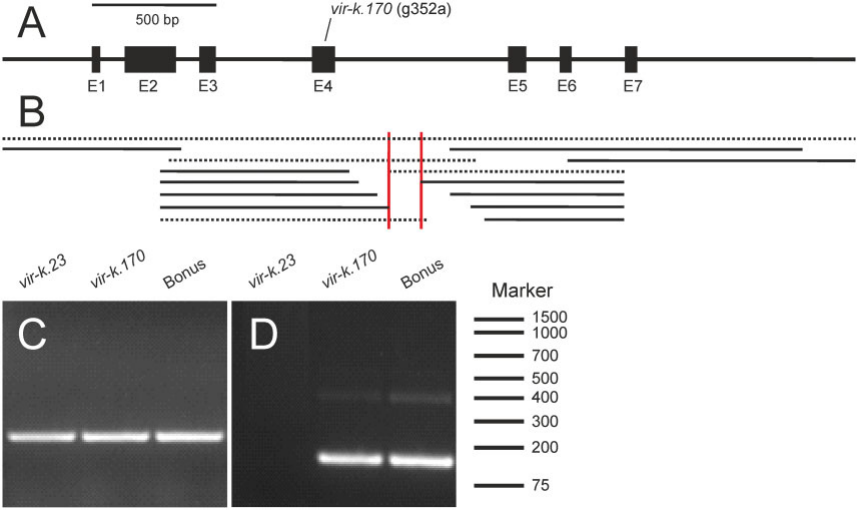
Gene structure of barley *Viridis-k*. A, The analyzed region of the *Viridis-k* gene consisting of seven exons; E1-E7. The *vir-k.170* point mutation is located in E4. B, The *vir-k.23* mutation was mapped by PCR using genomic DNA as template. Gene regions that could and could not be amplified are indicated by filled or dashed lines, respectively. The mapping and linkage analyses suggested a large chromosomal rearrangement interrupting the gene in the region between the two red vertical bars. C and D, RT-PCR amplicons separated by agarose gel electrophoresis and stained with Midori Green. First strand cDNA synthesis was performed with total RNA isolated from *vir-k.23*, *vir-k.170* and the cultivar Bonus, followed by end-point PCR. C, Amplification of a 237-bp cDNA fragment using a forward primer located in exon 1 and a reverse primer located in exon 2. D, Amplification of a 152-bp cDNA fragment using a forward primer located in exon 5 and a reverse primer located in exon 7. The 152-bp fragment could not be amplified from *vir-k.23*.

Sanger sequencing identified a single base pair substitution, a G–A transition, in the *vir-k.170* mutant. The transition changed a GCC codon to an ACC codon, which results in an alanine to threonine substitution (Ala-118-Thr; Figure 4). The G to A transition is consistent with the types of mutations that have previously been reported in barley after sodium azide mutagenesis (Olsen et al., 1993), which is the treatment used to generate *vir-k.170*. In the *vir-k.23* mutant the gene failed to be amplified by PCR using primers at the extreme ends of the gene but the 5′ and 3′ portions could be amplified separately (Figure 5). This suggested that the *vir-k.23* mutation is a larger chromosomal rearrangement that is consistent with the possible mutations induced by X-rays (Randolph, 1950; Hagberg and Tjio, 1951).

To further characterize the mutants, we performed a reverse-transcription PCR (RT-PCR) analysis of the *FdC2* ortholog in *vir-k.23* and *vir-k.170* as well as the control Bonus, which is the cultivar that the mutants were induced in. As the *vir-k.23* mutant appeared to have a chromosomal break in the middle of the gene, we designed two primer pairs, one pair on either side of the hypothesized break, to test for the presence of transcripts. This revealed that the 5′ portion of the gene was transcribed in both the mutants as well as the control. However, no transcript was detected covering the 3′ portion in the *vir-k.23* mutant even though this region could be amplified from genomic DNA and must therefore be present somewhere in the genome. The *vir-k.170* mutant and Bonus accumulated RNA transcribed from the 3′ portion of the gene as expected (Figure 5).

### The *viridis-k* phenotype can be complemented in vivo by *Viridis-k* but not by *Fd1*

**Figure 6:**
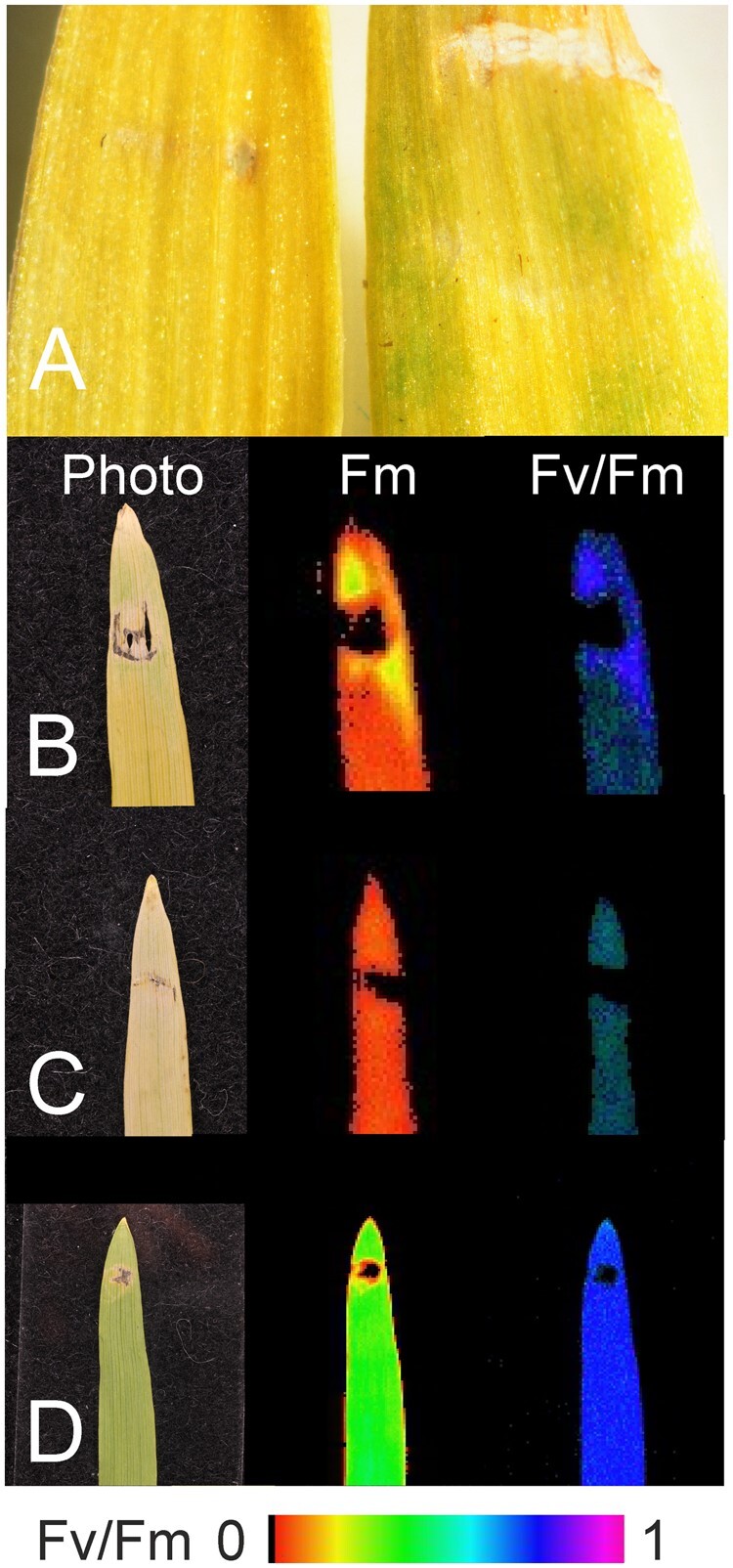
Complementation of the *vir-k.23* mutation by agroinfiltration. *A. tumefaciens* AGL0 harboring a plasmid containing either barley ferredoxin *Viridis-k* or ferredoxin *Fd1*, was infiltrated into seedling leaves of *vir-k.23*. Between 5 and 7 days postinfiltration, the leaves were inspected for presence of chlorophyll by visual inspection or by fluorescence using an imaging PAM. A, Green spots can be seen in a mutant seedling leaf injected with *Viridis-k* (right) but not with Fd1 (left). B, Infiltration with *Viridis-k*. Chlorophyll was formed around the injection site. The maximum chlorophyll a fluorescence (Fm) and the maximum quantum yield of photosystem II (Fv/Fm) are shown. A photo of the same leaf is shown to the left. C, Infiltration with Fd1. No chlorophyll could be detected. D, Photo and image of the mother cultivar Bonus infiltrated with *Viridis-k*.

**Figure 7:**
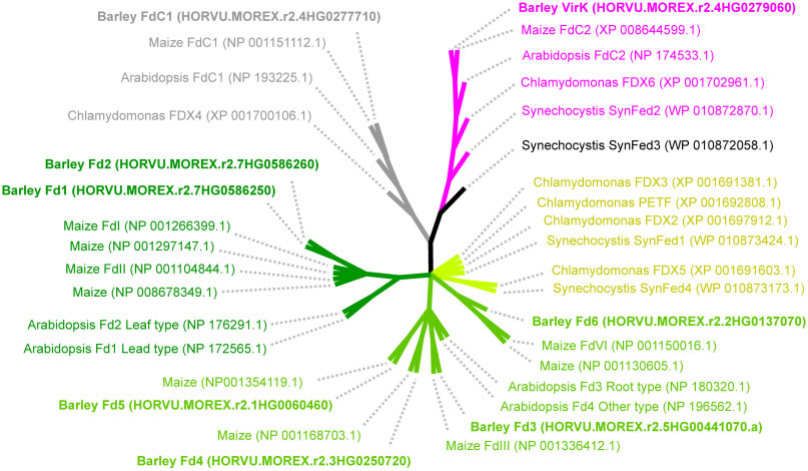
Maximum likelihood phylogenetic tree of plant-type ferredoxins. The eight barley ferredoxins are written in bold and used to identify ferredoxins by BLAST in maize, Arabidopsis, Chlamydomonas, and *Synechocystis* where ferredoxin isoforms have been previously characterized. The phylogeny is shown as an unrooted condensed bootstrap consensus tree generated in MEGA X and visualized using iTOL. Nodes with less than 50% bootstrap support were condensed. Branch lengths do not vary in bootstrap consensus trees generated in MEGA X and the tree should be interpreted as the maximum likelihood topology. Three main subtrees are apparent. These subtrees correspond to the canonical ferredoxins (dark green, light green, and lighter green) as well as the FdC1 (gray), and FdC2 (purple) types. The FdC2 subtree includes ferredoxins from all species while the FdC1 subtree does not contain a ferredoxin from *Synechocystis*. Canonical ferredoxins from plants have generally been classified as leaf-type and root-type. A leaf-type subtree of ferredoxins from plants is apparent (dark green) but the remaining canonical ferredoxins from plants (light green) do not form an obvious root-type subtree based on previously characterized ferredoxins. The main photosynthetic ferredoxins from Chlamydomonas and *Synechocystis* are PETF/FDX1 and SynFed1, respectively, located in the cluster indicated by lighter green.

To further confirm that the *viridis* phenotype was indeed due to disruption of the identified gene we set out to perform in vivo genetic complementation. Because of the lengthy process and genotype-specific requirements of performing stable transformations in barley (Harwood, 2014), we employed a transient expression approach based on agroinfiltration. This technique, has to the best of our knowledge, not previously been used to complement chlorophyll deficient mutants. To do this, we cloned full-length *Viridis-k* from cDNA obtained from the barley cultivar Bonus and inserted in a plant overexpression vector for *Agrobacterium tumefaciens* mediated transformation. As a control, the canonical photosynthetic ferredoxin *Fd1* was cloned into the same vector. The transient expression resulted in chlorophyll formation around the site of infiltration when *Viridis-k* was used but not *Fd1*. The successful complementation was best visualized with an imaging-PAM to reveal photosystem II chlorophyll fluorescence (Figure 6). This clearly showed presence of photosystem II bound chlorophyll, thus confirming that the suggested candidate gene, orthologous to Arabidopsis *FdC2*, is deficient in the *viridis-k* mutants and that *Viridis-k* is required for proper accumulation of chlorophyll in vivo. A search through the barley genome revealed eight plastid localized ferredoxins that are transcribed in the nucleus (Figure 7;Mayer et al., 2012; Colmsee et al., 2015; Mascher et al., 2017; Monat et al., 2019). The chlorophyll deficient phenotype of *viridis-k* mutants and the lack of complementation when leaf-type *Fd1* was used for agroinfiltration suggest that *Viridis-k* has a specific role that cannot be adequately performed by other ferredoxins in vivo.

### Presence of cyclase components in known mutants

**Figure 8:**
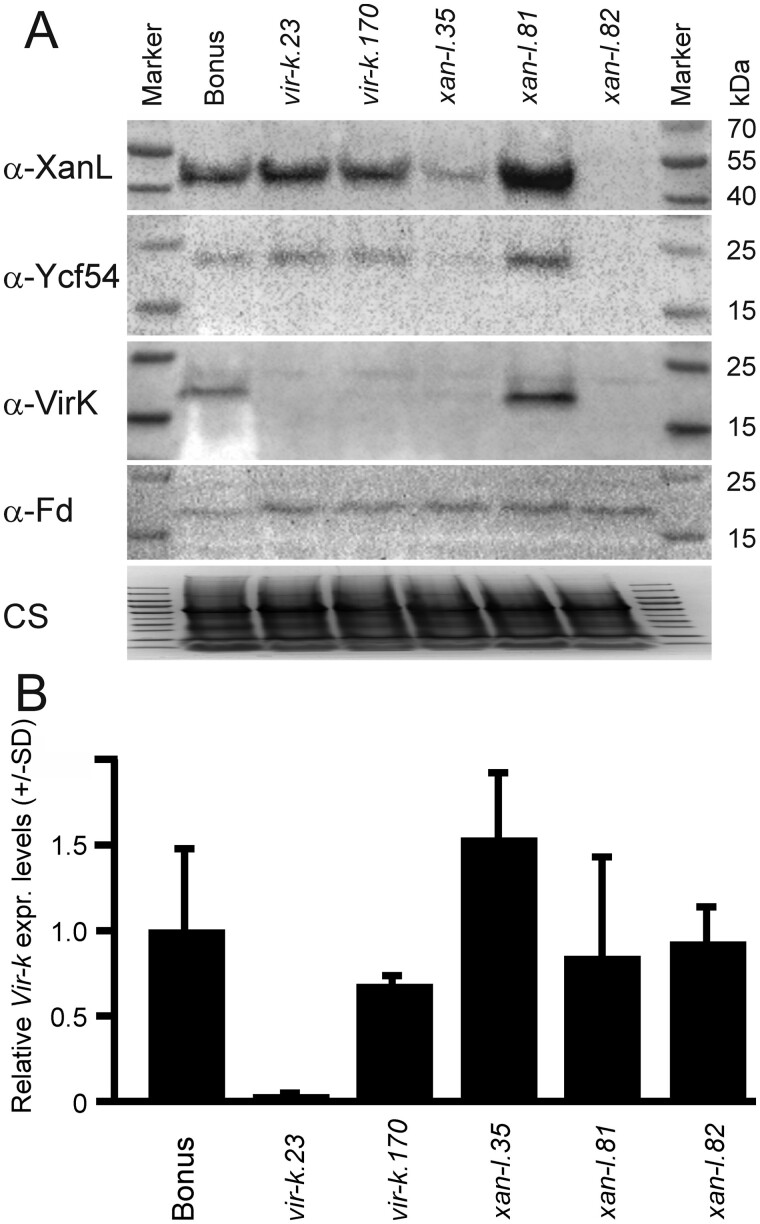
Immunoblots and RT-qPCR of barley mutants and the cultivar Bonus. A, Fifteen micrograms of total protein isolated from barley seedling leaves were separated by SDS-PAGE and transferred to PVDF membranes, which were incubated with antiserum raised against four different barley proteins as indicated in the figure. Coomassie-stained (CS) SDS-PAGE of the samples used in the immunoblot analyses showing that the same amount of protein has been loaded in each well. The CS image has been compressed. B, Total RNA was isolated and used for RT-qPCR analyses. The pair of primers used amplified the 5′ region of the *Viridis-k* transcript (Supplemental Table S2). The relative expression of *Viridis-k* compared to Bonus is shown. Only the transcript level in *vir-k.23* is significantly different (Supplemental Table S6).

Immunoblot analysis was performed on total protein extracts from barley cyclase mutants, *xan-l.35*, *xan-l.81*, and *xan-l.82* as well as *vir-k.23* and *vir-k.170*. The *xan-l.35* mutant has a point mutation and a leaky phenotype producing small amounts of chlorophyll. The *xan-l.81* and *xan-l.82* mutants are both completely blocked at the cyclase step and have a single amino acid substitution and introduction of an early stop codon, respectively (Rzeznicka et al., 2005). Blots with antibodies against XanL showed a dramatic decrease of XanL in *xan-l.35*, while no XanL accumulated in *xan-l.82*. The *vir-k.23* and *vir-k.170* mutants accumulated XanL to similar levels as the control cultivar Bonus (Figure 8A). The same pattern was observed for immunoblots probed with antibodies raised against the cyclase-associated protein Ycf54, which shows that Ycf54 does not accumulate in the absence of XanL and that the levels of Ycf54 increase with increasing levels of XanL (Figure 8A). Immunoblots using an antibody that recognizes canonical ferredoxin show the presence in all lines. However, immunoblots using antibodies raised against VirK showed that no VirK protein accumulates in the *vir-k.23* mutant, whereas trace amounts were seen in the *vir-k.170* mutant (Figure 8A). Interestingly, VirK accumulation also increases with increasing XanL accumulation in the *xantha-l* mutants. That is, VirK decreased to hardly detectable levels in *xan-l.35* and failed to accumulate in the *xan-l.82* mutant that lacks XanL. The effect is apparently posttranslational since quantitative RT-PCR (RT-qPCR) analysis showed similar levels of *Viridis-k* mRNA in all mutants except *vir-k.23*, which has a large chromosomal rearrangement (Figure 8B). That VirK accumulation is apparently dependent on cellular accumulation of XanL indicates an important in vivo interaction.

### VirK can function as an electron donor to the cyclase reaction

**Figure 9:**
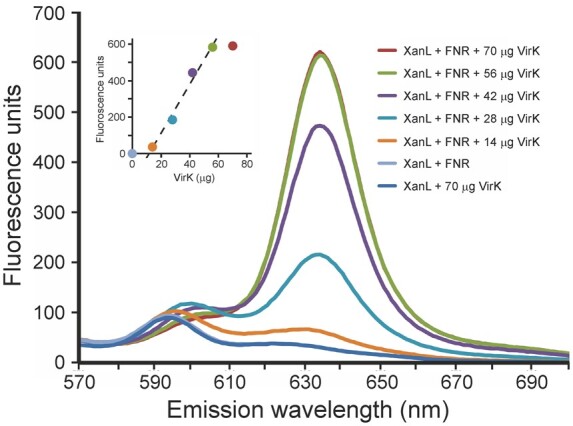
Enzymatic activity of recombinant VirK in combination with XanL and FNR. The XanL (40 µg) used was coexpressed with Ycf54, and the FNR (13 µg) was of the root type. Assay volumes were 30 µL. Product formation increased linearly with the amount of added VirK in the range of 14–56 µg (inset, *y* = 13.6*x* – 163, *R*^2^ = 0.986, *P* = 0.0071). No activity was observed when VirK or FNR was omitted from the assay.

As the XanL is a ferredoxin-dependent enzyme and the VirK isoform is clearly associated with the cyclase reaction in vivo, it seems likely that VirK is the main electron donor to the enzyme. To test if VirK could provide electrons to drive the cyclase reaction we produced recombinant VirK and used it as the electron donor for cyclase enzyme activity assays using recombinant XanL. The results clearly showed that VirK can drive the cyclase reaction with electrons provided by NADPH via FNR (Figure 9). Formation of the product, protochlorophyllide, increased with increasing VirK concentrations until saturation was reached, clearly showing that VirK can donate electrons to the cyclase enzyme. No product was formed if VirK was omitted from the reaction.

## Discussion

Plants and other photosynthetic organisms have a rich variety of ferredoxins. A search of the barley nuclear genome revealed eight ferredoxins with a chloroplast transit peptide. A comparison of these to ferredoxins of maize (*Zea mays* L.) and Arabidopsis as well as the green alga *Chlamydomonas reinhardtii* and the cyanobacterium *Synechocystis* PCC6803 shows three classes of ferredoxins (Figure 7). Both of the plant ferredoxin isoforms with C-terminal extensions, FdC1 and FdC2, form well-supported subtrees. The FdC2 subtree contains VirK as well as orthologs in all species including *Synechocystis*. This is consistent with a highly conserved and ancient function which is supported by the essential nature of the gene in plants as well as cyanobacteria (Cassier-Chauvat and Chauvat, 2014; Schorsch et al., 2018). The FdC1 subtree does not contain a representative from *Synechocystis* but the Chlamydomonas FDX4 ortholog clusters into this group.

The canonical ferredoxin isoforms from plants were first characterized as “photosynthetic” and “nonphotosynthetic” but now are generally referred to as “leaf-type” and “root-type,” respectively. The separation into different categories was based on early studies which found that maize contains isoforms located primarily in green tissue (FdI and FdII) which are regulated by light and an isoform found primarily in roots (FdIII) where photosynthesis does not occur (Hase et al., 1991). Another apparent difference between these two categories is that the leaf-type ferredoxins generally have a more negative redox potential than the root type. Thus, the midpoint potential for NADPH is in-between that of leaf- and root-type ferredoxins. The midpoint potential of ferredoxin isoforms is likely an important biochemical property for predicting in vivo function since leaf-type ferredoxins are reduced by photosystem I in order to reduce NADP^+^ to NADPH via FNR, while root-type ferredoxins are reduced by NADPH via FNR to support ferredoxin dependent metabolism in the absence of photosynthesis (Shinohara et al., 2017). The leaf-type ferredoxins form a clear subtree and both Arabidopsis and barley have two apparent leaf-type ferredoxins. Maize has four leaf-type ferredoxins, which is likely due to a need for additional specialization since maize has a C4 metabolism. Consistent with this, FdI and FdII have been previously characterized as being specific for bundle sheath cells and mesophyll cells, respectively (Kimata and Hase, 1989; Matsumura et al., 1999). Arabidopsis contains, in addition to the two leaf-type ferredoxins, two more canonical ferredoxins which were classified as a root-type and an “other” type based on divergent sequence and a much higher redox potential than the root-type (Hanke et al., 2004). Both of the fall into the same subtree which also contains three maize ferredoxins including the root type. Barley also has three ferredoxins in this subtree. A third subtree separate from the leaf- and root-type ferredoxins contains only representatives from barley and maize, both of which are monocots. One of these, maize FdVI, has been shown to be induced in roots by nitrate (Matsumura et al., 1997). Ferredoxins in this subtree may thus have a monocot-specific role in nitrogen assimilation.

The Chlamydomonas and *Synechocystis* ferredoxins, except for SynFed3, are clearly most closely related to canonical plant ferredoxins although they are divergent enough to not cluster within the leaf and root types. In Chlamydomonas, the main photosynthetic ferredoxin is PETF/FDX1 that has a midpoint potential of −398 mV (Terauchi et al., 2009) which is similar to that of Arabidopsis and maize leaf-type ferredoxins which range from −433 to −406 mV (Hase et al., 1991; Hanke et al., 2004). The Chlamydomonas FDX2 is most closely related to PETF/FDX1 but has a midpoint potential of −321 mV (Terauchi et al., 2009) which is similar to that of Arabidopsis and maize root-type ferredoxins which have midpoint potentials of −337 and −321 mV, respectively (Hase et al., 1991; Hanke et al., 2004). Based on the midpoint potentials, FDX2 may have similar functions to plant root-type ferredoxins in supporting ferredoxin-dependent metabolism in the absence of photosynthesis.

Mutants are invaluable tools for revealing molecular processes in vivo. The yellow mutants of vascular plants are chlorophyll deficient and have been explored to learn about genes and enzymes involved in chlorophyll biosynthesis. The biosynthetic step inhibited by a mutation is generally identified by accumulation of the substrate of the affected enzyme. However, a feedback mechanism prevents accumulation of chlorophyll biosynthetic intermediates unless the plants are fed with 5-aminolevulinic acid, which is the common precursor after the main regulatory step of the pathway. In this way, barley chlorophyll mutants were assigned to specific steps in the chlorophyll biosynthetic pathway (Gough, 1972). The *vir-k.23* and *vir-k.170* mutants were shown to be deficient in the cyclase reaction (Steccanella et al., 2015). In this study, we identified the *Viridis-k* locus as the gene orthologous to Arabidopsis *FdC2*, Chlamydomonas *FDX6*, and *Synechocystis SynFed2* encoding one of the two ferredoxin isoforms with C-terminal extension. Genetic mapping data identified a region containing 149 high-confidence genes including an *FdC2* ortholog, which appeared as the top candidate for several reasons. First, the known cyclase components are located in the chloroplast. Therefore, the gene product of the candidate gene should be localized to the chloroplast. The FdC2 ortholog was one of 32 gene products in the mapping interval with a predicted chloroplast transit peptide. Second, the cyclase reaction requires reducing power and was recently demonstrated to be a ferredoxin-dependent enzyme (Stuart et al., 2020) making the FdC2 ortholog the only chloroplast targeted candidate with an obvious functional association to the biochemistry of the cyclase enzyme. Previously, two mutants in the orthologous *FdC2* gene in rice had been isolated and were pale green due to decreased chlorophyll accumulation (Li et al., 2015; Zhao et al., 2015) but no further characterization or suggestions to their involvement in the cyclase reaction was reported. In addition, one would expect a gene required for chlorophyll biosynthesis to be highly conserved among oxygenic photosynthetic organisms. Recent studies on the evolution of photosystem I electron acceptors show that FdC2 orthologs are conserved and found in all green plastids (green algae and land plants) as well as cyanobacteria from which chloroplasts derive (Karpowicz et al., 2011; Pierella Karlusich et al., 2015; Pierella Karlusich and Carrillo, 2017).

Sanger sequencing identified a single base-pair mutation resulting in an Ala-118-Thr mutation in *vir-k.170*. The change from alanine to threonine is a drastic change since alanine is a small nonpolar amino acid while threonine is bulkier and polar. In addition, Ala-118 is highly conserved across C-type ferredoxins from a range of species including land plants, green algae, and cyanobacteria (Figure 4) which indicates that substitution of this residue is not well tolerated.

The *vir-k.23* mutant has a lesion in the *Viridis-k* gene. The mutation is consistent with a large-scale genomic rearrangement such as an inversion, a large insertion or a translocation spanning several million base pairs, and that one of the break points is within the *Viridis-k* gene. The 5′ and 3′ regions of the gene could be amplified by PCR in the *vir-k.23* mutant from genomic DNA but amplification across the central portion was not possible in the mutant while it was possible in the control cultivar Bonus, as well as in *vir-k.170.* This indicates that both ends of the gene are present in the genome but that they are no longer positioned such that they are in the proper orientation or appropriate distance from each other to allow amplification by PCR or production of a full-length transcript. Combined with the CAPS mapping data of *viridis-k.23* which showed a drop in the recombination frequency to zero over about 4 Mbp, the most likely scenario is a large-scale genomic rearrangement which would be consistent with the mutagen used since ionizing radiation such as X-rays are known to be able to induce double-stranded DNA breaks (Randolph, 1950; Hagberg and Tjio, 1951). The presence of a lesion in the middle of the gene was also supported by RT-PCR which showed that the *vir-k.23* mutant accumulates transcripts from the 5′ portion of the gene but not from the 3′ portion. Immunoblots against VirK show that the protein does not accumulate in the *vir-k.23* mutant which is expected considering that the gene is disrupted by a large structural mutation. Although not completely absent, VirK accumulation was reduced in the *vir-k.170* mutant, which suggests that the Ala-118-Thr substitution affects the in vivo stability of the protein. The mutation could affect either the physical stability of the protein and/or make it more prone to degradation by proteases. The level of *Viridis-k* mRNA in *vir-k.170* was not different from the mother cultivar Bonus (Figure 8).

Identification of multiple independent alleles is an efficient approach to validate a candidate gene. Over the years we have successfully used this approach where up to 215 alleles have been available; the *Eceriferum-cqu* locus involved in synthesis of epicuticular waxes as an example (Schneider et al., 2016). Unfortunately, only two *viridis-k* mutant alleles are available. To further support that we had identified the mutated gene in *viridis-k* mutants, we performed a transient expression of *Viridis-k* in seedling leaves of *vir-k.23* mutants. *A. tumefaciens* containing barley *Viridis-k* was infiltrated into the leaves. Typically, *Agrobacterium*-mediated infiltration is performed in dicot plants (Kapila et al., 1997; Wroblewski et al., 2005). After 5–7 days postinfiltration, chlorophyll formation could be seen around the site of infiltration conclusively demonstrating that the correct gene had been identified.

The *viridis-k* mutants are yellow, but trace amounts of chlorophyll can be detected (Steccanella et al., 2015). It is likely that this small amount of chlorophyll can be synthesized due to nonspecific action of other ferredoxins in the *vir-k.23* and *vir-k.170* mutants where VirK is absent or inactivated. Redundant cyclase activity supported by other ferredoxins might be expected since we recently showed that even leaf-type ferredoxin from spinach can drive the aerobic cyclase reaction in vitro using barley XanL (Stuart et al., 2020). Further, the aerobic cyclase, AcsF, of the purple bacterium *Rubrivivax gelatinosus* can be driven by ferredoxin from spinach and *Anabaena* (Chen et al., 2021). However, the *viridis-k* mutations are lethal and homozygous mutant seedlings die after approximately 2 weeks when the starch in the kernel is depleted. Thus, VirK plays a vital role in chlorophyll biosynthesis and its function cannot be meaningfully replaced by other ferredoxins in vivo. Additionally, *Viridis-k* is tightly connected to the cyclase enzyme system since we have shown that the steady state level of VirK is dependent on the level of XanL. This suggests an intimate connection between XanL and VirK in vivo. The same phenomenon has been observed previously with other enzymes involved in chlorophyll biosynthesis. For example, the magnesium chelatase, which inserts Mg^2+^ into protoporphyrin IX consists of three subunits encoded in barley by *Xantha-h*, *Xantha-g*, and *Xantha-f* (von Wettstein et al., 1974; Jensen et al., 1996). The *xantha-h* mutants that do not produce a XanH protein also fail to accumulate the XanG protein which is known to interact directly with XanH (Hansson et al., 1999; Lake et al., 2004; Lundqvist et al., 2010). The decreased accumulation of VirK is unlikely to be an indirect effect due to the lack of chlorophyll biosynthesis since VirK accumulates in the *xan-l.81* mutant which is also completely blocked at the cyclase step but does accumulate XanL. In contrast, the *viridis-k* mutants accumulate canonical ferredoxins but still have a chlorophyll deficient phenotype, and the *vir-k.23* mutant could be complemented by introducing *Viridis-k* but not leaf-type *Fd1*. Taken together with the fact that XanL is a ferredoxin dependent enzyme and that VirK can donate electrons to XanL in vitro, it is likely that VirK is the electron donor to the cyclase reaction in vivo and that the other ferredoxin isoforms offer little redundancy when it comes to supplying electrons to the cyclase enzyme.

An alternative function of *Synechocystis* Fed2 has recently been suggested (Schorsch et al., 2018). In their study, SynFed2 was suggested to be involved in iron homeostasis. An explanation consistent with their data and the results presented in this paper is that the observed phenotypes are due to a deficiency in chlorophyll biosynthesis. The cyanobacterial study made use of C-terminal truncations since complete deletion of the gene was lethal. In the absence of a complete loss of function mutant in cyanobacteria it is difficult to directly compare results, but the truncation mutants showed decreased growth and lower levels of chlorophyll accumulation.

As XanL is an iron-containing enzyme, one might speculate that the cyclase deficient phenotype in the *viridis-k* mutants is due to improper assembly of the diiron cluster of XanL. Improperly matured proteins tend to be degraded by the cell and this seems to be the case for improperly matured cyclase (Albus et al., 2012; Hollingshead et al., 2016; Chen and Hunter, 2020; Stuart et al., 2020). If the *viridis-k* mutations resulted in improper maturation of XanL we would expect to see decreased XanL levels in the *viridis-k* mutants that were not observed. In fact, the opposite was found—XanL is required for proper accumulation of VirK. Additionally, there was no apparent deficiency in the accumulation of canonical ferredoxin in the mutants which could also be expected if iron homeostasis was perturbed. The data are thus inconsistent with improperly matured XanL in the *viridis-k* mutants but are consistent with a function for VirK as the specific in vivo electron donor to the cyclase reaction.

## Materials and methods

### Plant material

The barley (*H. vulgare* L.) *vir-k.23* mutant was isolated in 1954 by X-ray mutagenesis of the cultivar Svalöv Weibull’s Bonus (Simpson and von Wettstein, 1980). The *xan-l.35* mutant was isolated in 1957 by ethyleneimine mutagenesis of Bonus while *xan-l.81* and *-l.82* mutants were isolated in 1975 by sodium azide mutagenesis of the *tigrina-d.12* mutant (Bonus genetic background) and screening for mutants that accumulated protochlorophyllide. The mutants were then backcrossed to Bonus in order to remove the *tigrina-d.12* mutation (Henningsen et al., 1993). Also in 1975, another mutant accumulating protochlorophyllide, originally named *xantha-83* was isolated which later turned out to be allelic to *vir-k.23* and thus renamed to *vir-k.170* (personal communications, Diter von Wettstein).

### Mapping population

An F_2_-mapping population was constructed by crossing the *vir-k.23* mutant to the cultivar Quench as the male parent. As the *vir-k.23* mutation is a recessive lethal mutation the female parent plants used were green and could be either homozygous wild-type or heterozygous for the *vir-k.23* mutation. Because of this, some F_1_ plants were homozygous wild-type and their offspring could not be used for mapping. To eliminate these individuals, the seeds of F_1_ plants were harvested on a per plant basis and one spike from each plant was grown in moist vermiculite for 9 days under a lab bench away from direct sunlight to check for segregation of the *vir-k.23* phenotype. Only seeds from F_1_ plants whose offspring segregated were kept for the mapping population. The F_2_-mapping population was grown under a lab bench away from direct sunlight. F_2_-plant material was collected after 9 days.

### Genomic DNA isolation

Genomic DNA for genotyping-by-sequencing library preparation or to be used as template for PCR was isolated by a modified CTAB protocol (Doyle, 1991). Between 100 and 200 mg leaf material was frozen under liquid nitrogen in screw cap tubes with two 4-mm glass beads and subsequently homogenized on a FastPrep 24 (MP Biomedicals, Santa Ana, CA) for 4 × 15 s at 4 m/s. Homogenized plant material was mixed with 1 mL 2 × CTAB buffer (2% [w/v] cetyltrimethylammonium bromide, 200 mM Tris–HCl pH 8, 20 mM EDTA, 1.4 M NaCl, 1% [w/v] polyvinylpyrrolidone [40 g/mol], 0.28 M β-mercaptoetanol) and incubated at 65°C for 30 min after which 800 µL chloroform:isoamylalcohol (24:1 [v/v]) was added and the samples were mixed on a rotary shaker for 15 min at room temperature. Samples were then centrifuged at 6,200*g* for 30 min at 4°C in order to separate the phases and 800 μL of the aqueous phase was transferred to a new tube and incubated at 37°C for 15 min with 50 μg RNase A. DNA was precipitated by addition of 560 μL isopropanol followed by centrifugation for 15 min at 15,700*g* and 4°C. The resulting pellet was then washed for 15 min in 76% (v/v) ethanol with 200 mM sodium acetate followed by a 5 min wash in 76% (v/v) ethanol with 10 mM ammonium acetate. After washing, the DNA pellet was allowed to dry at room temperature for 10 min prior to suspension in Low TE (10 mM Tris–HCl pH 8, 0.1 mM EDTA).

### Genotyping-by-sequencing library preparation

Genotyping-by-sequencing library preparation for 30 F_2_ seedlings was performed essentially as previously described (Poland et al., 2012) with minor modifications and using adaptor sequences as described in (Baird et al., 2008) with the modification that the P2 adaptors were modified to be compatible with *Msp*I digested DNA ends. Briefly, 200 ng gDNA per individual was digested with 8 units *Pst*I-HF (New England Biolabs) and 8 units *Msp*I (New England Biolabs) in 1 × NEB buffer 4 (New England Biolabs, Ipswich, MA) by incubation for 2 h at 37°C. Sample specific inline barcoded P1 adaptors as well as a common P2 Y-adaptor that have overhangs complementary to *Pst*I and *Msp*I, respectively, were then ligated to the digested gDNA using 200 units T4 DNA ligase (New England Biolabs) in a reaction mixture containing all 200 ng restriction digested gDNA, 1 mM ATP, 2.5 nM P1 adaptor, and 375 nM P2 adaptor in 1 × NEB buffer 4. The ligation reaction was incubated for 2 h at 22°C. At this point samples were pooled and concentrated to 60 μL using a Qiagen Quiaquick PCR purification spin column (Qiagen, Hilden, Germany) following manufacturer instructions. Next, 20 μL was used as template for a 200 μL PCR, split into 8 × 25 μL reactions using NEB NEXT Q5 Ultra II master mix (New England Biolabs). PCR enriches for DNA fragments containing both P1 and P2 adapters. Splitting of the PCR reaction mixture into multiple smaller reactions is done to minimize bias introduced by stochastic effects of PCR. After PCR the reactions were pooled and concentrated to 30 μL using a QiaQuick spin column (Qiagen) and run on a 1.25% (w/v) agarose 1× TAE gel at 100 V for 1 h. The region containing molecules in the size range of 450–650 bp was cut out in order to size select the DNA fragments. DNA was purified from the excised gel using a JetSorb gel extraction kit (Genomed, Warszawa, Poland). The JetSorb gel extraction was performed according to the manufacturer’s instructions with the modification that two extra washes with 80% (v/v) ethanol and 10 mM Tris–HCl pH 7.5 were added and the DNA was eluted in 60 μL Qiagen buffer EB. Sequencing was performed on an Illumina HiSeq 2500 with 125 cycles of paired-end sequencing with Illumina version 4 chemistry.

### Genotyping-by-sequencing data analysis

Raw reads were demultiplexed using the process_radtags component of the STACKS (version 1.40) pipeline (Catchen et al., 2011). Phenotypes and index sequences for the F_2_ individuals in the sequencing libraries can be found in Supplemental Table S1. Process_radtags produced four files for each individual, two files with paired reads, and two files with unpaired read. The four files for each individual were combined into a single file for each individual. Reads were then aligned to the barley reference genome (Mascher et al., 2017) using BWA mem (version 0.7.13) to produce sam files (Li and Durbin, 2010). Reads aligned to the reference genome were then further processed through the stacks pipeline to output a list of SNPs in variant call format (.vcf).

The vcf output file was further analyzed using TASSEL (version 5.2.31) in order to locate SNPs that were associated with the *viridis* phenotype in our mapping population (Bradbury et al., 2007). The vcf file was loaded in TASSEL and sorted followed by filtering SNPs such that they needed to be present in 20 individuals and have a minimum allele frequency of 0.4 and a maximum allele frequency of 0.6 (an allele has an expected frequency of 0.5 in an F_2_ population). Next, numerical values for phenotypes were assigned to the plants by setting wild-type to “0” and mutants to “100”. The General Linear Model (GLM) analysis was run with default settings except that “run permutations” was set to 10 and “Bi-allelic sites only” was selected since an SNP should have only two alleles in the mapping population. The output from the GLM analysis was then visualized to identify the genomic region linked to the mutant phenotype.

### CAPS genotyping and genetic mapping

Additional F_2_ individuals were genotyped using CAPS markers designed for SNPs discovered during genotyping-by-sequencing. For all markers, PCR was performed in 20 μL reactions using RedExtract-N-Amp (Sigma, St. Louis, MO, USA) following manufacturer’s instructions. PCR product was then digested with restriction enzymes (New England Biolabs) specific for one allele of the SNP to be analyzed followed by separation on a 2% (w/v) agarose 1 × TAE gel run for 22 min at 100 V. PCR primers used can be found in Supplemental Table S2. Five CAPS markers were tested located on chromosome 4H at 8.7 Mbp (4 units *Xba*I, Quench allele uncut), 9.7 Mbp (1 unit *Bts*CI, Quench allele cut), 10 Mbp (1 unit *Nco*I-HF, Quench allele cut), 13.7 Mbp (1 unit *Dde*I, Quench allele uncut), and 16.1 Mbp (1 unit *Nhe*I, Quench allele uncut). The recombination frequency between the SNPs and the *viridis-k* phenotype was calculated using a maximum likelihood approach (Fisher and Balmukand, 1928) and converted to genetic map units (cM) using the Kosambi mapping function (Kosambi, 1943).

### PCR and sequencing of the *Viridis-k* gene

Sequencing of the *Viridis-k* gene was performed on overlapping PCR amplicons by Eurofins Genomics on an ABI 3730XL DNA Analyzer. PCR was run with either Phusion high fidelity DNA polymerase (New England Biolabs) or RedExtract-N-Amp Plant Tissue PCR Kit (Sigma) following manufacturer’s guidelines. The PCR products were cleaned up using Illustra ExoProStar 1-Step (Cytiva, Marlborough, MA) following manufacturer’s instructions except if unspecific bands were produced in which case the PCR products were run on a 2% (w/v) agarose 1× TAE gel for 30 min at 100 V. The correct bands were then cut out of the gel and cleaned up with NucleoSpin Gel and PCR clean-up columns (Macherey-Nagel, Bethlehem, PA) before sequencing.

### RNA isolation, RT-PCR, and RT-qPCR

Plants were grown in vermiculite for 9 days under a lab bench away from direct sunlight and 300 mg material from a single leaf was harvested and immediately frozen in liquid nitrogen. Leaf material was homogenized under liquid nitrogen in a porcelain mortar. After homogenization total RNA was isolated using TRIzol reagent (Invitrogen Carlsbad, CA). Residual DNA was removed by treating total RNA preparations with DNase I (Thermo Scientific, Waltham, MA) after which cDNA was synthesized using 0.5 µg RNA per 20 µL reaction using RevertAid Reverse transcriptase (Thermo Scientific). The cDNA from *vir-k.23*, *vir-k.170* and Bonus were used for RT-PCR to detect *Viridis-k* transcripts and cDNA from Bonus was used for cloning of wild-type genes used in other experiments using Phusion high fidelity DNA polymerase (New England Biolabs).

For gene expression analysis using RT-qPCR, one leaf of three different plants was collected for RNA extraction per each biological replicate. Three technical replicates were performed on each biological sample. Total RNA was isolated using the RNeasy Plant Mini Kit (Qiagen) following the manufacturer’s instructions. RNA samples were treated by DNase on the columns using RNase-Free DNase Set (Qiagen). RNA integrity was checked on a 2% (w/v) agarose gel. The concentration and purity of the extracted RNA were measured spectrophotometrically. cDNA was synthesized from 1 µg of total RNA using the RevertAid First Strand cDNA synthesis kit (Thermo Scientific) following the manufacturer’s instructions. Transcript levels were measured by using the SsoAdvanced Universal SYBR Green Supermix (Bio-Rad, Hercules, CA). Each reaction contained 20× diluted cDNA and primers at a final concentration of 0.25 µM. Samples were run on a CFX384TM Real-Time System (Bio-Rad) using the following thermal cycling conditions: 95°C for 2 min, followed by 40 cycles of 95°C for 10 s and 60°C for 10 s. The used pair of primers amplified the 5′ region of the *Viridis-k* transcript (Supplemental Table S2). For normalization, the housekeeping gene E3 ubiquitin-protein ligase *UPL6* gene (HORVU1Hr1G023480) was used. Results were expressed using the ΔCt calculation method (Schmittgen and Livak, 2008).

### In vivo genetic complementation by agroinfiltration

Full-length coding sequences of *Viridis-k* (MLOC_37911) and *Fd1* (MLOC_6135) were amplified from cDNA and cloned into the overexpression vector pK7WG2 (Karimi et al., 2002) by Gateway cloning (Invitrogen) to generate pK7WG2HvFdC2 and pK7WG2HvFd1. The pK7WG2 constructs were transformed into *A. tumefaciens* strain AGL0. Seeds of *vir-k.23* were planted in vermiculite, watered, and placed in a cold room (4°C), giving the seeds time to imbibe water and thereby synchronize germination. After 48 h the seeds were moved to a climate chamber (16/8 h of light/dark, 26.5°C, 35% relative humidity, and 8,900 Lux). Bacteria were inoculated into LB media (10 g/L tryptone, 5 g/L yeast extract, and 10 g/L NaCl, pH 7) containing streptomycin (2 mg/mL). Cultures were incubated at 25°C with shaking for 48 h. New cultures were prepared by transferring bacteria from the two start cultures to two new cultures to OD_600_ of 0.1 in LB media containing streptomycin (2 mg/mL) and acetosyringone (200 μM). Cultures were incubated overnight at 25°C with shaking. Bacteria were pelleted by centrifugation (7,400*g* for 40 min). Supernatants were discarded and pellets were resuspended in MM buffer (10 mM 2-(N-morpholino) ethanesulfonic acid pH 5.7 and 10 mM MgCl_2_) containing 200 μM acetosyringone. The bacteria were washed by a second centrifugation (7,400*g* for 40 min). Supernatant was discarded and pellet resuspended to an OD_600_ of 1.1 in MM buffer containing 300 μM acetosyringone. Cultures were then incubated for 1–2 h at room temperature. Ascorbic acid to 20 mM was added to cultures immediately prior to infiltration. Bacteria were delivered into the abaxial side of the leaf by first creating a small scratch with a needle and then pressure infiltrating bacteria with a needleless syringe. Infiltrated seedlings were placed in dark overnight, under a plastic hood to maintain humidity. The next day the infiltrated seedlings were moved to a climate chamber (same settings as before) for visual observations.

### Chlorophyll fluorescence detection

Chlorophyll fluorescence was detected using an Imaging PAM M-series Maxi Version Chlorophyll Fluorometer (Heinz Walz, Effeltrich, Germany). The leaves were dark-adapted for a minimum of 10 min before analysis. The leaves were cut off directly prior to measurements, placed with adaxial side up under microscope slides to be held in position. Data were analyzed using ImagingWin (version2.47) software (Heinz Walz).

### Immunoblots

Polyclonal rabbit antibodies against barley VirK were generated by Agrisera using recombinant VirK as the antigen. Other antibodies used were raised against barley proteins XanL (Bollivar et al., 2014), Ycf54 (Stuart et al., 2020), and purified ferredoxin (Andersen et al., 1992). Plants for total protein extraction were grown in vermiculite for 9 days under a lab bench away from direct sunlight and 200 mg tissue was snap frozen in liquid nitrogen in 2-mL screw cap vials with two 4 mm glass beads and 10 µL 2.5% (w/v) PMSF in isopropanol. Plant material was homogenized by grinding in a FastPrep 24 at 4 m/s for 10 s repeated 10 times with 4 min under liquid nitrogen between cycles to prevent material from thawing. Next, 500 µL protein extraction buffer (12 M urea, 2% [w/v] SDS, 100 mM DTT, 100 mM Tris–HCl pH 8, and 10 mM EDTA pH 8) was added, briefly heated to 60°C, and mixed by running four more cycles in the FastPrep 24. Samples were then incubated at 99°C and 1,400 rpm for 30 min in a Thermomixer comfort (Eppendorf, Hamburg, Germany) followed by centrifugation for 10 min at 16,000*g*. The supernatant was transferred to fresh tubes and centrifuged for another 30 min. The final supernatant was saved as the total protein extract. SDS-polyacrylamide gel (PAGE) and immunoblots were performed as described previously (Stuart et al., 2020).

### Recombinant protein production and cyclase activity assay

Recombinant XanL was prepared as described previously (Stuart et al., 2020). The coding sequence of *Viridis-k* without the transit peptide as predicted by ChloroP (Emanuelsson et al., 1999) was codon optimized and cloned into pET15b by GenScript Biotech Corporation to generate pET15bHvFdC2. *Escherichia coli* BL21(DE3) transformed with pET15bHvFdC2 was inoculated to an OD_600_ of 0.1 in 250 mL LB containing 100 µg/mL ampicillin and placed in an incubator at 20°C and 200 rpm. After 1 h the temperature was set down to 15°C. Once the culture reached an OD_600_ of 0.3, IPTG was added to a final concentration of 1 mM and solid FeSO_4_ was added to a final concentration of 2 mM. After 24 h another 2 mM equivalent of solid FeSO_4_ was added and the culture was grown for an additional 36 h before the cells were harvested by centrifugation and stored at −80°C until use. For protein purification a cell pellet was resuspended to 50 mL in binding buffer (20 mM Tris–HCl pH 8, 500 mM NaCl, 20 mM imidazole, and 5 mM DTT) supplemented with 3 M urea as well as a few grains of DNase I and lysozyme. The cell suspension was passed through a french press three times at 12.4 MPa after which the lysate was centrifuged 10 min at 48,384*g*. The supernatant was loaded on two 1 mL HisTrap FF crude (Cytiva) columns connected in series and washed four times with 15 mL binding buffer supplemented with decreasing concentrations of urea. The urea concentration was decreased from 3 M to no urea in 1 M increments. The column was then washed with 15 mL wash buffer (20 mM Tris–HCl pH 8, 500 mM NaCl, 40 mM imidazole, and 5 mM DTT) followed by elution with 20 mM Tris–HCl pH 8, 500 mM NaCl, 750 mM imidazole, and 5 mM DTT. The VirK protein was desalted over a NAP-10 (Cytiva) column into 50 mM Tris–HCl pH 8 with 1 mM DTT, after which 87% (v/v) glycerol was added to a final concentration of 15% (v/v). The VirK protein was aliquoted and stored at −80°C until use.

The coding sequence of barley root isoform of FNR (HvRFNR, MLOC_6838) was cloned from cDNA without the chloroplast transit peptide as predicted by ChloroP into the vector pDEST17 by Gateway cloning to produce pDEST17HvRFNR. *E. coli* Rosetta (DE3)pLysS transformed with pDEST17HvRFNR was inoculated to an OD_600_ of 0.1 into 250 mL LB supplemented with 100 µg/mL ampicillin and 25 µg/mL chloramphenicol and grown at 25°C and 200 rpm until the culture reached an OD_600_ of 0.4, after which protein expression was induced by adding IPTG to a final concentration of 1 mM. Cells were harvested by centrifugation the next morning after 12–16 h of growth postinduction and cell pellets were frozen at −80°C until use. A cell pellet corresponding to 250 mL culture was resuspended to 20 mL in binding buffer supplemented with 100 µg/mL lysozyme after which cells were disrupted by sonication on ice for a total of 4 min at 50% output. The lysate was centrifuged 8 min at 48,384*g*, after which the supernatant was loaded onto a 1 mL HisTrap FF crude column. The column was washed with 5 mL binding buffer followed by 10 mL wash buffer. The recombinant HvRNFR was eluted with 20 mM Tris–HCl pH 8, 500 mM NaCl, 250 mM imidazole, and 5 mM DTT and desalted over a NAP-10 column into buffer consisting of 1 mM DTT, 25 mM MgCl_2_, 1 mM EDTA, 20 mM Tricine, 10 mM HEPES, and adjusted to pH 8.1 with NaOH. After desalting, 87% (v/v) glycerol was added to a final concentration of 15% (v/v) and the protein was aliquoted and frozen at −80°C until use. Cyclase assays were run as described previously (Stuart et al., 2020) except that the above recombinant HvRFNR and VirK were used instead of spinach FNR and ferredoxin from Sigma. MPE was extracted from a *bchE* mutant of *Rhodobacter capsulatus* as previously described (Gough et al., 2007). A measurement series consisted of six enzymatic reactions with different amounts (0–70 µg) of added VirK but constant amounts of XanL (40 µg) and HvRNFR (13 µg) in total volumes of 30 µL containing 20 mM Tricine and 10 mM HEPES pH 8.1, 1 mM EDTA, 25 mM MgCl_2_, 1 mM DTT, 10 mM glucose-6-phosphate, 0.03 units/µL glucose-6-phosphate dehydrogenase, 0.5 mM NADPH, 90 µg/µL catalase, 0.026% (v/v) Triton X-100, and 10 µM MPE. Assays were incubated in the dark at 30°C and 750 rpm for 1 h in a Thermomixer comfort (Eppendorf). Assays were stopped by the addition of 80% (v/v) acetone with 0.32% (v/v) NH_3_ and centrifuged for 5 min at 29,000 × *g* to pellet precipitated proteins. Formation of protochlorophyllide was measured using an RF-5301 PC spectrofluorometer (Shimadzu. Kyoto, Japan) with an excitation wavelength of 440 nm and an emission spectrum between 570 and 700 nm with slit widths of 10 nm for both excitation and emission. Product formation was estimated as the relative fluorescence emission at 634 nm for samples minus the emission for the negative control sample. Linear regression was performed with the lm() function in R (version 3.6.1).

### Phylogenetic analysis

Ferredoxin sequences for Arabidopsis, maize, Chlamydomonas, and *Synechocystis* were retrieved by BLAST against the NCBI Protein reference sequences database with an E value cutoff of 10^−20^ for a match using each of the barley ferredoxins as queries. In order to avoid bias from erroneous prediction of chloroplast transit peptides all sequences were N-terminally trimmed to the shortest *Synechocystis* ferredoxin after removal of the initiator methionine as these do not provide a phylogenetic signal. Sequences were aligned using ClustalW as implemented in MEGA-X (version 10.2.5). The alignment in FASTA format can be found in Supplemental File S1. MEGA-X was used to determine the best fitting maximum likelihood model after which a maximum likelihood tree was inferred using the LG +G model with 16 rate categories and gamma parameter equal to 1.6872. Node support was determined using 1,000 bootstrap replicates. Nodes with less than 50% support were condensed in the bootstrap consensus tree. The tree was visualized as an unrooted tree using iTOL (Letunic and Bork, 2021). The tree in Newick format can be found in Supplemental File S2.

## Accession numbers

Sequence data from this article can be found in the NCBI Sequence Read Archive (https://www.ncbi.nlm.nih.gov/sra) under accession no. PRJNA686392.

## Supplemental data

The following materials are available in the online version of this article.

**Supplemental Table S1**. Phenotype and index sequence for F_2_ individuals included in the genotyping-by-sequencing libraries for mapping of the *Viridis-k* gene.

**Supplemental Table S2**. List of used DNA oligonucleotides.

**Supplemental Table S3**. Positions and genetic distances of markers to the *Viridis-k* gene.

**Supplemental Table S4**. Ct values from RT-qPCR of the 5′ region of the *Viridis-k* gene.

**Supplemental Table S5**. Ct values from RT-qPCR of the housekeeping gene E3 ubiquitin-protein ligase *UPL6* (HORVU1Hr1G023480).

**Supplemental Table S6**. Calculations of relative expression of *Viridis-k* in the five mutants and their mother cultivar Bonus.

**Supplemental Data Set S1**. List of genes in the mapped interval for *Viridis-k*.

**Supplemental File S1**. Multiple sequence alignment of ferredoxin peptide sequences used for constructing phylogenetic relations in Figure 7.

**Supplemental File S2**. Phylogenetic tree (Figure 7) in Newick format.

## Supplementary Material

koab150_Supplementary_Data

## Notes

D.S., D.B., M.H., and P.E.J. designed the experiments; D.S., H.M.Y., M.S., and S.Z. performed the experiments; D.S., D.B., and M.H. analyzed the data; D.S., D.B., M.H., and P.E.J. wrote the article. All authors read and approved the final article.

The author responsible for distribution of materials integral to the findings presented in this article in accordance with the policy described in the Instructions for Authors (https://academic.oup.com/plcell) is: Mats Hansson (mats.hansson@biol.lu.se).

## References

[koab150-B1] Albus CA , Salinas A, Czarnecki O, Kahlau S, Rothbart M, Thiele W, Lein W, Bock R, Grimm B, Schottler MA (2012) LCAA, a novel factor required for magnesium protoporphyrin monomethylester cyclase accumulation and feedback control of aminolevulinic acid biosynthesis in tobacco. Plant Physiol 160: 1923–1939 23085838 10.1104/pp.112.206045 PMC3510121

[koab150-B2] Andersen B , Koch B, Scheller HV (1992) Structural and functional analysis of the reducing side of photosystem I. Physiol Plant 84: 154–161

[koab150-B3] Baird NA , Etter PD, Atwood TS, Currey MC, Shiver AL, Lewis ZA, Selker EU, Cresko WA, Johnson EA (2008) Rapid SNP discovery and genetic mapping using sequenced RAD markers. PLoS One 3: e3376 18852878 10.1371/journal.pone.0003376 PMC2557064

[koab150-B4] Banerjee R , Jones JC, Lipscomb JD (2019) Soluble methane monooxygenase. Annu Rev Biochem 88: 409–431 30633550 10.1146/annurev-biochem-013118-111529

[koab150-B5] Berthold DA , Stenmark P (2003) Membrane-bound diiron carboxylate proteins. Annu Rev Plant Biol 54: 497–517 14503001 10.1146/annurev.arplant.54.031902.134915

[koab150-B6] Bollivar D , Braumann I, Berendt K, Gough SP, Hansson M (2014) The Ycf54 protein is part of the membrane component of Mg-protoporphyrin IX monomethyl ester cyclase from barley (*Hordeum vulgare* L). FEBS J 281: 2377–2386 24661504 10.1111/febs.12790

[koab150-B7] Bradbury PJ , Zhang Z, Kroon DE, Casstevens TM, Ramdoss Y, Buckler ES (2007) TASSEL: software for association mapping of complex traits in diverse samples. Bioinformatics 23: 2633–2635 17586829 10.1093/bioinformatics/btm308

[koab150-B8] Bryant DA , Hunter CN, Warren MJ (2020) Biosynthesis of the modified tetrapyrroles-the pigments of life. J Biol Chem 295: 6888–6925 32241908 10.1074/jbc.REV120.006194 PMC7242693

[koab150-B9] Cassier-Chauvat C , Chauvat F (2014) Function and regulation of ferredoxins in the cyanobacterium, synechocystis PCC6803: recent advances. Life (Basel) 4: 666–680 25387163 10.3390/life4040666 PMC4284462

[koab150-B10] Catchen JM , Amores A, Hohenlohe P, Cresko W, Postlethwait JH (2011) *Stacks*: building and genotyping loci *de novo* from short-read sequences. G3 1: 171–182 22384329 10.1534/g3.111.000240 PMC3276136

[koab150-B11] Chen GE , Hunter CN (2020) Protochlorophyllide synthesis by recombinant cyclases from eukaryotic oxygenic phototrophs and the dependence on Ycf54. Biochem J 477: 2313–2325 32469391 10.1042/BCJ20200221 PMC7319587

[koab150-B12] Chen GE , Canniffe DP, Hunter CN (2017) Three classes of oxygen-dependent cyclase involved in chlorophyll and bacteriochlorophyll biosynthesis. Proc Natl Acad Sci USA 114: 6280–6285 28559347 10.1073/pnas.1701687114 PMC5474816

[koab150-B13] Chen GE , Adams NBP, Jackson PJ, Dickman MJ, Hunter CN (2021) How the O_2_-dependent Mg-protoporphyrin monomethyl ester cyclase forms the fifth ring of chlorophylls. Nat Plants 7: 365–375 33731920 10.1038/s41477-021-00876-3 PMC7610348

[koab150-B14] Chen GE , Canniffe DP, Barnett SFH, Hollingshead S, Brindley AA, Vasilev C, Bryant DA, Hunter CN (2018) Complete enzyme set for chlorophyll biosynthesis in *Escherichia coli*. Sci Adv 4: eaaq1407 29387799 10.1126/sciadv.aaq1407 PMC5787379

[koab150-B15] Colmsee C , Beier S, Himmelbach A, Schmutzer T, Stein N, Scholz U, Mascher M (2015) BARLEX - the barley draft genome explorer. Mol Plant 8: 964–966 25804976 10.1016/j.molp.2015.03.009

[koab150-B16] Doyle J (1991) DNA protocols for plants taxonomy. *In*Hewitt GM, Johnston AWB, Young JPW eds, Molecular Techniques. Springer, Berlin, Heidelberg, Germany, pp 283–293

[koab150-B17] Emanuelsson O , Nielsen H, von Heijne G (1999) ChloroP, a neural network-based method for predicting chloroplast transit peptides and their cleavage sites. Protein Sci 8: 978–984 10338008 10.1110/ps.8.5.978 PMC2144330

[koab150-B18] Fisher RA , Balmukand B (1928) The estimation of linkage from the offspring of selfed heterozygotes. J Genet 20: 79–92

[koab150-B19] Gough S (1972) Defective synthesis of porphyrins in barley plastids caused by mutation in nuclear genes. Biochim Biophys Acta 286: 36–54 4659262 10.1016/0304-4165(72)90086-4

[koab150-B20] Gough SP , Rzeznicka K, Peterson Wulff R, da Cruz Francisco J, Hansson A, Jensen PE, Hansson M (2007) A new method for isolating physiologically active Mg-protoporphyrin monomethyl ester, the substrate of the cyclase enzyme of the chlorophyll biosynthetic pathway. Plant Physiol Biochem 45: 932–936 17949988 10.1016/j.plaphy.2007.09.001

[koab150-B21] Guan X , Chen S, Voon CP, Wong KB, Tikkanen M, Lim BL (2018) FdC1 and leaf-type ferredoxins channel electrons from photosystem I to different downstream electron acceptors. Front Plant Sci 9: 410. 29670639 10.3389/fpls.2018.00410 PMC5893904

[koab150-B22] Hagberg A , Tjio JH (1951) Cytological studies on some X-ray mutants of barley. An Estac Exp Aula Dei 2: 149–167

[koab150-B23] Hanke G , Mulo P (2013) Plant type ferredoxins and ferredoxin-dependent metabolism. Plant Cell Environ 36: 1071–1084 23190083 10.1111/pce.12046

[koab150-B24] Hanke GT , Kimata-Ariga Y, Taniguchi I, Hase T (2004) A post genomic characterization of Arabidopsis ferredoxins. Plant Physiol 134: 255–264 14684843 10.1104/pp.103.032755 PMC316305

[koab150-B25] Hansson A , Kannangara CG, von Wettstein D, Hansson M (1999) Molecular basis for semidominance of missense mutations in the XANTHA-H (42-kDa) subunit of magnesium chelatase. Proc Natl Acad Sci USA 96: 1744–1749 9990095 10.1073/pnas.96.4.1744 PMC15580

[koab150-B26] Harwood WA (2014) A protocol for high-throughput *Agrobacterium*-mediated barley transformation. Methods Mol Biol 1099: 251–260 24243209 10.1007/978-1-62703-715-0_20

[koab150-B27] Hase T , Kimata Y, Yonekura K, Matsumura T, Sakakibara H (1991) Molecular cloning and differential expression of the maize ferredoxin gene family. Plant Physiol 96: 77–83 16668188 10.1104/pp.96.1.77 PMC1080715

[koab150-B28] Henningsen KW , Boynton JE, Wettstein DV (1993) Mutants at *Xantha* and *Albina* Loci in Relation to Chloroplast Biogenesis in Barley (*Hordeum vulgare* L.). The Royal Danish Academy of Sciences and Letters, Copenhagen

[koab150-B29] Hollingshead S , Kopecna J, Armstrong DR, Bucinska L, Jackson PJ, Chen GE, Dickman MJ, Williamson MP, Sobotka R, Hunter CN (2016) Synthesis of chlorophyll-binding proteins in a fully segregated *Dycf54* strain of the cyanobacterium *Synechocystis* PCC 6803. Front Plant Sci 7: 292 27014315 10.3389/fpls.2016.00292 PMC4794507

[koab150-B30] Jensen PE , Willows RD, Petersen BL, Vothknecht UC, Stummann BM, Kannangara CG, von Wettstein D, Henningsen KW (1996) Structural genes for Mg-chelatase subunits in barley: *Xantha-f*, *-g* and *-h*. Mol Gen Genet 250: 383–394 8602155 10.1007/BF02174026

[koab150-B31] Kapila J , De Rycke R, Van Montagu M, Angenon G (1997) An *Agrobacterium*-mediated transient gene expression system for intact leaves. Plant Sci 122: 101–108

[koab150-B32] Karimi M , Inzé D, Depicker A (2002) GATEWAY vectors for *Agrobacterium*-mediated plant transformation. Trends Plant Sci 7: 193–195 11992820 10.1016/s1360-1385(02)02251-3

[koab150-B33] Karpowicz SJ , Prochnik SE, Grossman AR, Merchant SS (2011) The GreenCut2 resource, a phylogenomically derived inventory of proteins specific to the plant lineage. J Biol Chem 286: 21427–21439 21515685 10.1074/jbc.M111.233734 PMC3122202

[koab150-B34] Kimata Y , Hase T (1989) Localization of ferredoxin isoproteins in mesophyll and bundle sheath cells in maize leaf. Plant Physiol 89: 1193–1197 16666683 10.1104/pp.89.4.1193 PMC1055995

[koab150-B35] Kolton M , Keren I, Shevtsov S, Shaya F, Peled-Zehavi H, Danon A, Ostersetzer-Biran O (2011) Plastidic redox switches: ferredoxins as novel RNA-binding proteins. Endocyt Cell Res 21: 1–18

[koab150-B36] Kosambi DD (1943) The estimation of map distances from recombination values. Ann Eugen 12: 172–175

[koab150-B37] Lake V , Olsson U, Willows RD, Hansson M (2004) ATPase activity of magnesium chelatase subunit I is required to maintain subunit D *in vivo*. Eur J Biochem 271: 2182–2188 15153108 10.1111/j.1432-1033.2004.04143.x

[koab150-B38] Letunic I , Bork P (2021) Interactive tree of life (iTOL) v5: an online tool for phylogenetic tree display and annotation. Nucleic Acids Res 23: 127–128 10.1093/nar/gkab301 PMC8265157 33885785

[koab150-B39] Li C , Hu Y, Huang R, Ma X, Wang Y, Liao T, Zhong P, Xiao F, Sun C, Xu Z, et al. (2015) Mutation of *FdC2* gene encoding a ferredoxin-like protein with C-terminal extension causes yellow-green leaf phenotype in rice. Plant Sci 238: 127–134 26259181 10.1016/j.plantsci.2015.06.010

[koab150-B40] Li H , Durbin R (2010) Fast and accurate long-read alignment with Burrows-Wheeler transform. Bioinformatics 26: 589–595 20080505 10.1093/bioinformatics/btp698 PMC2828108

[koab150-B41] Lundqvist J , Elmlund H, Wulff RP, Berglund L, Elmlund D, Emanuelsson C, Hebert H, Willows RD, Hansson M, Lindahl M, et al (2010) ATP-induced conformational dynamics in the AAA+ motor unit of magnesium chelatase. Structure 18: 354–365 20223218 10.1016/j.str.2010.01.001

[koab150-B42] Mascher M , Gundlach H, Himmelbach A, Beier S, Twardziok SO, Wicker T, Radchuk V, Dockter C, Hedley PE, Russell J, et al. (2017) A chromosome conformation capture ordered sequence of the barley genome. Nature 544: 427–433 28447635 10.1038/nature22043

[koab150-B43] Matsumura T , Sakakibara H, Nakano R, Kimata Y, Sugiyama T, Hase T (1997) A nitrate-inducible ferredoxin in maize roots. Genomic organization and differential expression of two nonphotosynthetic ferredoxin isoproteins. Plant Physiol 114: 653–660 9193097 10.1104/pp.114.2.653 PMC158349

[koab150-B44] Matsumura T , Kimata-Ariga Y, Sakakibara H, Sugiyama T, Murata H, Takao T, Shimonishi Y, Hase T (1999) Complementary DNA cloning and characterization of ferredoxin localized in bundle-sheath cells of maize leaves. Plant Physiol 119: 481–488 9952443 10.1104/pp.119.2.481 PMC32124

[koab150-B45] Mayer KF , Waugh R, Brown JW, Schulman A, Langridge P, Platzer M, Fincher GB, Muehlbauer GJ, Sato K, Close TJ, et al (2012) A physical, genetic and functional sequence assembly of the barley genome. Nature 491: 711–716 23075845 10.1038/nature11543

[koab150-B46] Monat C , Padmarasu S, Lux T, Wicker T, Gundlach H, Himmelbach A, Ens J, Li C, Muehlbauer GJ, Schulman AH, et al (2019) TRITEX: chromosome-scale sequence assembly of Triticeae genomes with open-source tools. Genome Biol 20: 284 31849336 10.1186/s13059-019-1899-5 PMC6918601

[koab150-B47] Olsen O , Wang X, von Wettstein D (1993) Sodium azide mutagenesis: preferential generation of A⋅T → G⋅C transitions in the barley *Ant18* gene. Proc Natl Acad Sci USA 90: 8043–8047 8367460 10.1073/pnas.90.17.8043 PMC47284

[koab150-B48] Pierella Karlusich JJ , Carrillo N (2017) Evolution of the acceptor side of photosystem I: ferredoxin, flavodoxin, and ferredoxin-NADP^+^ oxidoreductase. Photosynth Res 134: 235–250 28150152 10.1007/s11120-017-0338-2

[koab150-B49] Pierella Karlusich JJ , Ceccoli RD, Graña M, Romero H, Carrillo N (2015) Environmental selection pressures related to iron utilization are involved in the loss of the flavodoxin gene from the plant genome. Genome Biol Evol 7: 750–767 25688107 10.1093/gbe/evv031 PMC5322553

[koab150-B50] Poland JA , Brown PJ, Sorrells ME, Jannink JL (2012) Development of high-density genetic maps for barley and wheat using a novel two-enzyme genotyping-by-sequencing approach. PLoS One 7: e32253 22389690 10.1371/journal.pone.0032253 PMC3289635

[koab150-B51] Porra RJ , Schafer W, Katheder I, Scheer H (1995) The derivation of the oxygen atoms of the 13^1^-oxo and 3-acetyl groups of bacteriochlorophyll *a* from water in *Rhodobacter sphaeroides* cells adapting from respiratory to photosynthetic conditions: evidence for an anaerobic pathway for the formation of *iso*cyclic ring E. FEBS Lett 371: 21–24 7664876 10.1016/0014-5793(95)00854-3

[koab150-B52] Randolph LF (1950) Cytological and phenotypical effects induced in maize by x-rays and the bikini test able atomic bomb. J Cell Physiol Suppl 35: 103–117 15436532 10.1002/jcp.1030350408

[koab150-B53] Rzeznicka K , Walker CJ, Westergren T, Kannangara CG, von Wettstein D, Merchant S, Gough SP, Hansson M (2005) *Xantha-l* encodes a membrane subunit of the aerobic Mg-protoporphyrin IX monomethyl ester cyclase involved in chlorophyll biosynthesis. Proc Natl Acad Sci USA 102: 5886–5891 15824317 10.1073/pnas.0501784102 PMC556314

[koab150-B54] Schmittgen TD , Livak KJ (2008) Analyzing real-time PCR data by the comparative *C*_T_ method. Nat Protoc 3: 1101–1108 18546601 10.1038/nprot.2008.73

[koab150-B55] Schneider LM , Adamski NM, Christensen CE, Stuart DB, Vautrin S, Hansson M, Uauy C, von Wettstein-Knowles P (2016) The *Cer-cqu* gene cluster determines three key players in a β-diketone synthase polyketide pathway synthesizing aliphatics in epicuticular waxes. J Exp Bot 67: 2715–2730 26962211 10.1093/jxb/erw105 PMC4861019

[koab150-B56] Schorsch M , Kramer M, Goss T, Eisenhut M, Robinson N, Osman D, Wilde A, Sadaf S, Brückler H, Walder L, et al (2018) A unique ferredoxin acts as a player in the low-iron response of photosynthetic organisms. Proc Natl Acad Sci USA 115: E12111–E12120 30514818 10.1073/pnas.1810379115 PMC6304933

[koab150-B57] Shinohara F , Kurisu G, Hanke G, Bowsher C, Hase T, Kimata-Ariga Y (2017) Structural basis for the isotype-specific interactions of ferredoxin and ferredoxin: NADP^+^ oxidoreductase: an evolutionary switch between photosynthetic and heterotrophic assimilation. Photosynth Res 134: 281–289 28093652 10.1007/s11120-016-0331-1

[koab150-B58] Sievers F , Wilm A, Dineen D, Gibson TJ, Karplus K, Li W, Lopez R, McWilliam H, Remmert M, Söding J, et al (2011) Fast, scalable generation of high-quality protein multiple sequence alignments using Clustal Omega. Mol Syst Biol 7: 539 21988835 10.1038/msb.2011.75 PMC3261699

[koab150-B59] Simpson DJ , von Wettstein D (1980) Macromolecular physiology of plastids XIV. *Viridis* mutants in barley: genetic, fluoroscopic and ultrastructural characterisation. Carlsberg Res Commun 45: 283–314

[koab150-B60] Steccanella V , Hansson M, Jensen PE (2015) Linking chlorophyll biosynthesis to a dynamic plastoquinone pool. Plant Physiol Biochem 97: 207–216 26480470 10.1016/j.plaphy.2015.10.009

[koab150-B61] Stuart D , Sandström M, Youssef HM, Zakhrabekova S, Jensen PE, Bollivar DW, Hansson M (2020) Aerobic barley Mg-protoporphyrin IX monomethyl ester cyclase is powered by electrons from ferredoxin. Plants 9: 1157 10.3390/plants9091157 PMC7570240 32911631

[koab150-B62] Tagawa K , Arnon DI (1962) Ferredoxins as electron carriers in photosynthesis and in the biological production and consumption of hydrogen gas. Nature 195: 537–543 14039612 10.1038/195537a0

[koab150-B63] Tanaka R , Tanaka A (2007) Tetrapyrrole biosynthesis in higher plants. Annu Rev Plant Biol 58: 321–346 17227226 10.1146/annurev.arplant.57.032905.105448

[koab150-B64] Terauchi AM , Lu SF, Zaffagnini M, Tappa S, Hirasawa M, Tripathy JN, Knaff DB, Farmer PJ, Lemaire SD, Hase T, et al (2009) Pattern of expression and substrate specificity of chloroplast ferredoxins from *Chlamydomonas reinhardtii*. J Biol Chem 284: 25867–25878 19586916 10.1074/jbc.M109.023622 PMC2757988

[koab150-B65] Tottey S , Block MA, Allen M, Westergren T, Albrieux C, Scheller HV, Merchant S, Jensen PE (2003) *Arabidopsis* CHL27, located in both envelope and thylakoid membranes, is required for the synthesis of protochlorophyllide. Proc Natl Acad Sci USA 100: 16119–16124 14673103 10.1073/pnas.2136793100 PMC307702

[koab150-B66] Walker CJ , Mansfield KE, Smith KM, Castelfranco PA (1989) Incorporation of atmospheric oxygen into the carbonyl functionality of the protochlorophyllide isocyclic ring. Biochem J 257: 599–602 2930469 10.1042/bj2570599 PMC1135620

[koab150-B67] Wiesselmann M , Hebecker S, Borrero-de Acuña JM, Nimtz M, Bollivar DW, Jänsch L, Moser J, Jahn D (2020) Mg-protoporphyrin IX monomethyl ester cyclase from *Rhodobacter capsulatus*: Radical SAM-dependent synthesis of the isocyclic ring of bacteriochlorophylls. Biochem J 477: 4635–4654 33211085 10.1042/BCJ20200761

[koab150-B68] von Wettstein D , Kahn A, Nielsen OF, Gough S (1974) Genetic regulation of chlorophyll synthesis analyzed with mutants in barley. Science 184: 800–802 17783474 10.1126/science.184.4138.800

[koab150-B69] Voss I , Goss T, Murozuka E, Altmann B, McLean KJ, Rigby SE, Munro AW, Scheibe R, Hase T, Hanke GT (2011) FdC1, a novel ferredoxin protein capable of alternative electron partitioning, increases in conditions of acceptor limitation at photosystem I. J Biol Chem 286: 50–59 20966083 10.1074/jbc.M110.161562 PMC3013009

[koab150-B70] Wroblewski T , Tomczak A, Michelmore R (2005) Optimization of *Agrobacterium*-mediated transient assays of gene expression in lettuce, tomato and *Arabidopsis*. Plant Biotechnol J 3: 259–273 17173625 10.1111/j.1467-7652.2005.00123.x

[koab150-B71] Zhao J , Qiu Z, Ruan B, Kang S, He L, Zhang S, Dong G, Hu J, Zeng D, Zhang G, et al(2015) Functional inactivation of putative photosynthetic electron acceptor ferredoxin C2 (FdC2) induces delayed heading date and decreased photosynthetic rate in rice. PLoS One 10: e0143361 26598971 10.1371/journal.pone.0143361 PMC4657970

