# Supplementary material for: Barley Viridis-k links an evolutionarily conserved C-type ferredoxin to chlorophyll biosynthesis
Source: Plant Cell. 2021 May 29;33(8):2834–49. doi: 10.1093/plcell/koab150 (PMC8408499; doi:10.1093/plcell/koab150)
Supplement: koab150_Supplementary_Data [file koab150_supplementary_data.zip › tpc.00018.2021-s02.docx]

**Supplemental Table S1.** Phenotype and index sequence for F_2_ individuals included in the genotyping-by-sequencing libraries for mapping of the *Viridis-k* gene.

| \| **F_2_ Individual** \| **Sequencing Library** \| **Index sequence** \| **Phenotype** \| \| --- \| --- \| --- \| --- \| \|  \|  \|  \|  \| \| MH84-134-1 \| 1 \| ACATAGG \| Wild Type \| \| MH84-103-3 \| 1 \| CGAACTG \| Wild Type \| \| MH84-106-3 \| 1 \| GTACGTG \| Wild Type \| \| MH84-115-3 \| 1 \| TCTCTCA \| *vir-k.23* \| \| MH84-119-3 \| 1 \| ACGATTG \| Wild Type \| \| MH84-120-3 \| 1 \| AGTCTGT \| Wild Type \| \| MH84-122-3 \| 1 \| ATTACGG \| Wild Type \| \| MH84-126-3 \| 1 \| CACTAGT \| Wild Type \| \| MH84-130-3 \| 1 \| CATCGTA \| *vir-k.23* \| \| MH84-133-3 \| 1 \| CGACAAT \| Wild Type \| \| MH84-134-3 \| 1 \| GACCACA \| Wild Type \| \| MH84-103-5 \| 1 \| GGTAACT \| *vir-k.23* \| \| MH84-106-5 \| 1 \| GTCTTGG \| Wild Type \| \| MH84-119-5 \| 1 \| TACGCTA \| Wild Type \| \| MH84-120-5 \| 1 \| TCAGGAA \| Wild Type \| \| MH84-134-2 \| 2 \| ACATAGG \| Wild Type \| \| MH84-103-4 \| 2 \| CGAACTG \| *vir-k.23* \| \| MH84-106-4 \| 2 \| GTACGTG \| Wild Type \| \| MH84-115-4 \| 2 \| TCTCTCA \| Wild Type \| \| MH84-119-4 \| 2 \| ACGATTG \| Wild Type \| \| MH84-120-4 \| 2 \| AGTCTGT \| *vir-k.23* \| \| MH84-122-4 \| 2 \| ATTACGG \| Wild Type \| \| MH84-126-4 \| 2 \| CACTAGT \| Wild Type \| \| MH84-130-4 \| 2 \| CATCGTA \| *vir-k.23* \| \| MH84-133-4 \| 2 \| CGACAAT \| *vir-k.23* \| \| MH84-134-4 \| 2 \| GACCACA \| Wild Type \| \| MH84-122-5 \| 2 \| GGTAACT \| *vir-k.23* \| \| MH84-126-5 \| 2 \| GTCTTGG \| Wild Type \| \| MH84-133-5 \| 2 \| TACGCTA \| Wild Type \| \| MH84-134-5 \| 2 \| TCAGGAA \| *vir-k.23* \| |
| --- | --- | --- | --- | --- | --- | --- | --- | --- | --- | --- | --- | --- | --- | --- | --- | --- | --- | --- | --- | --- | --- | --- | --- | --- | --- | --- | --- | --- | --- | --- | --- | --- | --- | --- | --- | --- | --- | --- | --- | --- | --- | --- | --- | --- | --- | --- | --- | --- | --- | --- | --- | --- | --- | --- | --- | --- | --- | --- | --- | --- | --- | --- | --- | --- | --- | --- | --- | --- | --- | --- | --- | --- | --- | --- | --- | --- | --- | --- | --- | --- | --- | --- | --- | --- | --- | --- | --- | --- | --- | --- | --- | --- | --- | --- | --- | --- | --- | --- | --- | --- | --- | --- | --- | --- | --- | --- | --- | --- | --- | --- | --- | --- | --- | --- | --- | --- | --- | --- | --- | --- | --- | --- | --- | --- | --- | --- | --- | --- |

**Supplemental Table S2.** List of DNA oligonucleotides used in this study for cloning, polymerase chain reaction, and Sanger sequencing.

| **Name** | **Sequence** | **Description** |
| --- | --- | --- |
| HvFdC2_F1 | GTGTACAAAGCCGTGAGCATTC | Forward primer for PCR and Sanger sequencing of the *Viridis-k* gene |
| HvFdC2_F2 | TCGTCCACCAGTTCGTCGT | Forward primer for PCR and Sanger sequencing of the *Viridis-k* gene |
| HvFdC2_F3 | GTAGGAAGAGAACGGATGTATGTG | Forward primer for PCR and Sanger sequencing of the *Viridis-k* gene |
| HvFdC2_F4 | CACAAGGTGACCGTCCACGA | Forward primer for PCR and Sanger sequencing of the *Viridis-k* gene |
| HvFdC2_F5 | CAAGCTTTGTGCGTCACAGT | Forward primer for PCR and Sanger sequencing of the *Viridis-k* gene |
| HvFdC2_F6 | GCGAGGGCCTGTTGTAAGTA | Forward primer for PCR and Sanger sequencing of the *Viridis-k* gene |
| HvFdC2_F7 | TCATCCATTTTGCTTCGTATGTAAC | Forward primer for PCR and Sanger sequencing of the *Viridis-k* gene |
| HvFdC2_F8 | AGGAAGAGAACGGATGTATGTGA | Forward primer for PCR and Sanger sequencing of the *Viridis-k* gene |
| HvFdC2_F9 | TATCACGTGAATCGGGGTGC | Forward primer for PCR and Sanger sequencing of the *Viridis-k* gene |
| HvFdC2_F10 | CCTACAGGCTTGCTTCGTGA | Forward primer for PCR and Sanger sequencing of the *Viridis-k* gene |
| HvFdC2_R1 | CAATGCGAGAGCGGAACGTA | Reverse primer for PCR and Sanger sequencing of the *Viridis-k* gene |
| HvFdC2_R2 | ATAGAGCACCCCGATTCACG | Reverse primer for PCR and Sanger sequencing of the *Viridis-k* gene |
| HvFdC2_R3 | ACTTCCTTCCATAAGCTTGGTC | Reverse primer for PCR and Sanger sequencing of the *Viridis-k* gene |
| HvFdC2_R4 | GGGTTACATACGAAGCAAAATGGAT | Reverse primer for PCR and Sanger sequencing of the *Viridis-k* gene |
| HvFdC2_R5 | AATGGAAAGACGGGCCGATT | Reverse primer for PCR and Sanger sequencing of the *Viridis-k* gene |
| HvFdC2_R6 | GACGTGTAAAACGCAGACCG | Reverse primer for PCR and Sanger sequencing of the *Viridis-k* gene |
| HvFdC2_R7 | GGTCCAACAGTCGAACCGAT | Reverse primer for PCR and Sanger sequencing of the *Viridis-k* gene |
| HvFdC2_R8 | CTGAACGGGATCATCTGACAGT | Reverse primer for PCR and Sanger sequencing of the *Viridis-k* gene |
| HvFdC2_R9 | ATTGCAGTACCGCACACAAAC | Reverse primer for PCR and Sanger sequencing of the *Viridis-k* gene |
| HvFdC2_R10 | GGAAGGAGCATGAAGATCGGA | Reverse primer for PCR and Sanger sequencing of the *Viridis-k* gene |
| RT-HvFdC2_F1 | AGTCCAAGGCCAGGATGG | Forward primer used for RT-PCR of *Viridis-k* 5' region |
| RT-HvFdC2_R1 | CGACGAACTGGTGGACGA | Reverse primer used for RT-PCR of *Viridis-k* 5' region |
| RT-HvFdC2_F2 | GCTATGCGTTGTTATGTGTTGG | Forward primer used for RT-PCR of *Viridis-k* 3' region |
| RT-HvFdC2_R2 | CTCGTCTCCCATTGCGAG | Reverse primer used for RT-PCR of *Viridis-k* 3' region |
| HvSNP_8693162_F | GCACCCTATTGTCAAAAGACCA | Forward primer for CAPS SNP at 8.6 Mbp |
| HvSNP_8693162_R | CCGTCCTGTTCCATCGATCA | Reverse primer for CAPS SNP at 8.6 Mbp |
| HvSNP_9708039_F | TTCACCTTTGTCTACACCAC | Forward primer for CAPS SNP at 9.7 Mbp |
| HvSNP_9708039_R | AGGACGAAGACAAACTATGC | Reverse primer for CAPS SNP at 9.7 Mbp |
| HvSNP_10033984_F | CTCGTCCTGAAACCCTAATCC | Forward primer for CAPS SNP at 10 Mbp |
| HvSNP_10033984_R | GCAGGTGTAAAGATAAGGCGAT | Reverse primer for CAPS SNP at 10 Mbp |
| HvSNP_13709787_F | GCGTCGGAACATTTGGTTGC | Forward primer for CAPS SNP at 13.7 Mbp |
| HvSNP_13709787_R | CTCATGGGGTGTTTGAGTGC | Reverse primer for CAPS SNP at 13.7 Mbp |
| HvSNP_16087547_F | CAGTACATGCGAGCCGATTTG | Forward primer for CAPS SNP at 16.1 Mbp |
| HvSNP_16087547_R | CAACTTGTTCTCTCGCCAATGG | Reverse primer for CAPS SNP at 16.1 Mbp |
| HvFdC2_GW_For_+cTP | GGGGACAAGTTTGTACAAAAAAGCAGGCTTCGAAGGAGATAGAACCATGGCGGCGTGCCCCGCCGCGAC | Forward primer for Gateway cloning of *Viridis-k* with cTP from cDNA |
| HvFdC2_GW_Rev | GGGGACCACTTTGTACAAGAAAGCTGGGTCTCCACCTCCGGATCACTCGTCTCCCATTGCGAGCTCTAGC | Forward primer for Gateway cloning of *Viridis-k* with cTP from cDNA |
| HvLFd1_GW_For_+cTP | GGGGACAAGTTTGTACAAAAAAGCAGGCTTCGAAGGAGATAGAACCATGGCCGCCGCACTGAGC | Forward primer for Gateway cloning of *HvLFd1* with cTP from cDNA |
| HvLFd1_GW_Rev | GGGGACCACTTTGTACAAGAAAGCTGGGTCTTATGCGGTGAGCTCCTCCTCCT | Forward primer for Gateway cloning of *HvLFd1* with cTP from cDNA |
| HvRFNR_GW_For_no_cTP | GGGGACAAGTTTGTACAAAAAAGCAGGCTTCCAGTCGAGCAAGAGCAAGGT | Forward primer for Gateway cloning of *HvRFNR* without cTP from cDNA |
| HvRFNR_GW_Rev | GGGGACCACTTTGTACAAGAAAGCTGGGTCCTAGTAAACCTCAACGTGCCATTG | Reverse primer for Gateway cloning of *HvRFNR* without cTP from cDNA |
| HvFdC2_qPCR_F1 | CCCAGGACCAGTACATTCTG | Forward primer used for RT-qPCR of *Viridis-k* 5' region |
| HvFdC2_qPCR_R1 | AGTCAGCCGATATTCCAAGG | Reverse primer used for RT-qPCR of *Viridis-k* 5' region |
| UPL6_qPCR_F | AACGATCTGCCTTCCTTGGA | Forward primer used for RT-qPCR of housekeeping gene |
| UPL6_qPCR_R | AAATCTGAGACGTCGCCCTT | Reverse primer used for RT-qPCR of housekeeping gene |

**Supplemental Table S3.** Chromosome 4H position and genetic distance to the *Viridis-k* gene of cleaved amplified polymorphic sequence markers used for narrowing the mapping interval.

| **Position**  **(Mbp)** | **Distance to**  ***Viridis-k* (cM)** | **Number F_2_ individuals genotyped** |
| --- | --- | --- |
| 8.7 | 6.7 | 285 |
| 9.7 | 0 | 285 |
| 10 | 0 | 190 |
| 13.7 | 0 | 285 |
| 16.1 | 2.1 | 281 |

**Supplemental Table S4.** Ct values from RT-qPCR with forward primer HvFdC2_qPCR_F1 and reverse primer HvFdC2_pPCR_R1, which targets the 5’ region of the *Viridis-k* gene. Three technical replicates on three biological replicates (three different plants) were performed for the five mutants and their mother cultivar Bonus. Ave, average; SD, standard deviation. This Supplemental Table supports Figure 8B.

|  | **Technical replicates** | | |  |  |
| --- | --- | --- | --- | --- | --- |
|  | **1** | **2** | **3** | **Ave** | **SD** |
| Bonus_1 | 21.76 | 21.45 | 21.67 | 21.63 | 0.1623 |
| Bonus_2 | 21.37 | 21.47 | 21.41 | 21.42 | 0.0511 |
| Bonus_3 | 24.22 | 22.23 | 23.94 | 23.46 | 1.0755 |
| xan-l.35_1 | 22.15 | 21.75 | 22.79 | 22.23 | 0.5231 |
| xan-l.35_2 | 21.83 | 22.00 | 21.72 | 21.85 | 0.1424 |
| xan-l.35_3 | 21.43 | 21.57 | 21.76 | 21.59 | 0.1678 |
| xan-l.81_1 | 23.06 | 21.25 | 26.50 | 23.60 | 2.6689 |
| xan-l.81_2 | 22.54 | 21.46 | 21.20 | 21.73 | 0.7117 |
| xan-l.81_3 | 23.16 | 23.40 | 23.09 | 23.22 | 0.1598 |
| xan-l.82_1 | 21.61 | 21.09 |  | 21.35 | 0.3659 |
| xan-l.82_2 | 24.00 | 22.53 | 21.58 | 22.70 | 1.2216 |
| xan-l.82_3 | 22.68 | 21.52 | 21.12 | 21.78 | 0.8087 |
| vir-k.23_1 | 28.01 | 29.41 | 27.67 | 28.36 | 0.9189 |
| vir-k.23_2 | 29.38 | 30.64 | 27.97 | 29.33 | 1.3355 |
| vir-k.23_3 | 28.21 | 28.57 | 27.67 | 28.15 | 0.4533 |
| vir-k.170_1 | 24.18 | 22.44 | 21.77 | 22.80 | 1.2406 |
| vir-k.170_2 | 24.12 | 21.65 | 21.47 | 22.42 | 1.4817 |
| vir-k.170_3 | 22.41 | 22.41 | 22.57 | 22.46 | 0.0904 |

**Supplemental Table S5.** Ct values from RT-qPCR with forward primer UPL6_qPCR_F and reverse primer UPL6_qPCR_R, which targets the housekeeping gene E3 ubiquitin-protein ligase UPL6 (HORVU1Hr1G023480). Three technical replicates on three biological replicates (three different plants) were performed for the five mutants and their mother cultivar Bonus. Ave, average; SD, standard deviation. This Supplemental Table supports Figure 8B.

|  | **Technical replicates** | | |  |  |
| --- | --- | --- | --- | --- | --- |
|  | **1** | **2** | **3** | **Ave** | **SD** |
| Bonus_1 | 25.40 | 25.88 | 26.05 | 25.78 | 0.3386 |
| Bonus_2 | 25.25 | 25.52 | 25.41 | 25.40 | 0.1346 |
| Bonus_3 | 25.21 | 25.78 | 25.80 | 25.60 | 0.3367 |
| xan-l.35_1 | 26.06 | 25.72 | 26.05 | 25.94 | 0.1902 |
| xan-l.35_2 | 26.22 | 26.44 | 26.69 | 26.45 | 0.2358 |
| xan-l.35_3 | 25.80 | 25.97 | 26.18 | 25.98 | 0.1888 |
| xan-l.81_1 | 25.81 | 26.30 | 26.66 | 26.26 | 0.4259 |
| xan-l.81_2 | 25.49 | 25.34 | 27.59 | 26.14 | 1.2612 |
| xan-l.81_3 | 25.61 | 25.60 | 25.26 | 25.49 | 0.2011 |
| xan-l.82_1 | 24.69 | 24.77 | 25.31 | 24.92 | 0.3382 |
| xan-l.82_2 | 25.05 | 25.37 | 26.96 | 25.80 | 1.0224 |
| xan-l.82_3 | 25.38 | 25.46 | 26.15 | 25.66 | 0.4218 |
| vir-k.23_1 | 25.59 | 29.30 | 25.85 | 26.92 | 2.0736 |
| vir-k.23_2 | 25.99 | 26.08 | 26.30 | 26.12 | 0.1596 |
| vir-k.23_3 | 26.02 | 25.92 | 26.03 | 25.99 | 0.0616 |
| vir-k.170_1 | 25.39 | 25.74 | 26.18 | 25.77 | 0.3975 |
| vir-k.170_2 |  | 25.38 | 25.53 | 25.46 | 0.1008 |
| vir-k.170_3 | 25.45 | 26.08 | 25.63 | 25.72 | 0.3259 |

**Supplemental Table S6.** Calculations of relative expression of *Viridis-k* in the five mutants and their mother cultivar Bonus using values from Supplemental Tables S4 and S5. Significant differences were tested by a two-sided t-test. SD. standard deviation. This Supplemental Table supports Figure 8B.

|  | **F1xR1** | **House-**  **keeping** | **Subtraction** | **2^ΔCt** | **1/x** | **Average** | **Relative Bonus** | **Relative Bonus** | **SD** | ***p* (t-test)** |
| --- | --- | --- | --- | --- | --- | --- | --- | --- | --- | --- |
| Bonus_1 | 21.63 | 25.78 | -4.15 | 0.0563 | 17.78 | 12.64 | 1.00 | 1.4061 | 0.4665 |  |
| Bonus_2 | 21.42 | 25.40 | -3.98 | 0.0634 | 15.77 |  |  | 1.247 |  |  |
| Bonus_3 | 23.46 | 25.60 | -2.13 | 0.2282 | 4.38 |  |  | 0.3467 |  |  |
| xan-l.35_1 | 22.23 | 25.94 | -3.71 | 0.0762 | 13.12 | 19.48 | 1.54 | 1.0379 | 0.3707 | 0.268 |
| xan-l.35_2 | 21.85 | 26.45 | -4.60 | 0.0412 | 24.27 |  |  | 1.9199 |  |  |
| xan-l.35_3 | 21.59 | 25.98 | -4.40 | 0.0475 | 21.06 |  |  | 1.6659 |  |  |
| xan-l.81_1 | 23.60 | 26.26 | -2.66 | 0.1586 | 6.31 | 10.80 | 0.85 | 0.4989 | 0.5871 | 0.797 |
| xan-l.81_2 | 21.73 | 26.14 | -4.41 | 0.0470 | 21.26 |  |  | 1.6816 |  |  |
| xan-l.81_3 | 23.22 | 25.49 | -2.27 | 0.2072 | 4.83 |  |  | 0.3817 |  |  |
| xan-l.82_1 | 21.35 | 24.92 | -3.57 | 0.0841 | 11.89 | 11.73 | 0.93 | 0.9406 | 0.2023 | 0.851 |
| xan-l.82_2 | 22.70 | 25.80 | -3.09 | 0.1173 | 8.52 |  |  | 0.6742 |  |  |
| xan-l.82_3 | 21.78 | 25.66 | -3.89 | 0.0677 | 14.78 |  |  | 1.1692 |  |  |
| vir-k.23_1 | 28.36 | 26.92 | 1.45 | 2.7317 | 0.37 | 0.23 | 0.02 | 0.0290 | 0.0083 | 0.041 |
| vir-k.23_2 | 29.33 | 26.12 | 3.20 | 9.2171 | 0.11 |  |  | 0.0086 |  |  |
| vir-k.23_3 | 28.15 | 25.99 | 2.16 | 4.4720 | 0.22 |  |  | 0.0177 |  |  |
| vir-k.170_1 | 22.80 | 25.77 | -2.97 | 0.1274 | 7.85 | 8.54 | 0.68 | 0.6211 | 0.0575 | 0.384 |
| vir-k.170_2 | 22.42 | 25.46 | -3.04 | 0.1216 | 8.22 |  |  | 0.6503 |  |  |
| vir-k.170_3 | 22.46 | 25.72 | -3.25 | 0.1048 | 9.55 |  |  | 0.7551 |  |  |
