# Supplementary material for: Barley Viridis-k links an evolutionarily conserved C-type ferredoxin to chlorophyll biosynthesis
Source: Plant Cell. 2021 May 29;33(8):2834–49. doi: 10.1093/plcell/koab150 (PMC8408499; doi:10.1093/plcell/koab150)
Supplement: koab150_Supplementary_Data [file koab150_supplementary_data.zip › tpc.00018.2021-s05.pdf]

Barley *Viridis-k* Links an Evolutionarily Conserved C-Type Ferredoxin to Chlorophyll Biosynthesis

David Stuart, Malin Sandström, Helmy M. Youssef, Shakhira Zakhrabekova, Poul Erik Jensen, David Bollivar, Mats Hansson

Corresponding author: Mats Hansson, mats.hansson@biol.lu.se

Review timeline:

**TPC2021-RA-00018**	Submission received:	Jan. 16, 2021
	1st Decision:	Feb. 12, 2021 *revision requested*
**TPC2021-RA-00018R1**	1st Revision received:	April 14, 2021
	2nd Decision:	May 10, 2021 *acceptance pending, sent to science editor*
	Final acceptance:	May 21, 2021

REPORT: (The report shows the major requests for revision and author responses. Minor comments for revision and miscellaneous correspondence are not included. The original format may not be reflected in this compilation, but the reviewer comments and author responses are not edited, except to correct minor typographical or spelling errors that could be a source of ambiguity.)

TPC2021-RA-00018 1st Editorial decision – *revision requested* **Feb. 12, 2021**

We have received reviews of your manuscript entitled "Barley *Viridis-k* links an evolutionary conserved C-type ferredoxin to chlorophyll biosynthesis." Thank you for submitting your best work to The Plant Cell. The editorial board agrees that the work you describe is substantive, falls within the scope of the journal, and may become acceptable for publication, pending revision and potential re-review.

We ask you to pay attention to the following points in preparing your revision: Below are the comments from three expert reviewers, all of whom support communication of this work but recommend changes to improve the accessibility of the work to a general audience. Please address all of the reviewer's queries and suggestions. No additional experimental work should be required, unless you already have data and/or wish to provide new data to address questions raised by Reviewer 1. In your revision, please address concerns

1) to address the technical issues and methodology presentation raised by Reviewer 2,

2) to tighten up the results and discussion to avoid overlap, and

3) to address the nomenclature and ortholog issues raised by Reviewer 3.

-- Reviewer comments:

[Reviewer comments shown below along with author responses]

TPC2021-RA-00018R1 1st Revision received **April 14, 2021**

Reviewer comments and **author responses**:

<u>Editor's comments:</u>

No additional experimental work should be required, unless you already have data and/or wish to provide new data to address questions raised by Reviewer 1.

RESPONSE: An RT-qPCR analysis was performed as suggested by Reviewer 1.

In your revision, please address concerns 1) to address the technical issues and methodology presentation raised by Reviewer 2

RESPONSE: Done as suggested.

2) to tighten up the results and discussion to avoid overlap

RESPONSE: Done to the best of our ability.

3) to address the nomenclature and ortholog issues raised by Reviewer 3.

RESPONSE: Has been addressed.

In your revised manuscript, please note the following:

The Plant Cell now requires authors to complete and submit an author revisions checklist upon submission of a revised manuscript. The aim of the checklist is to aid authors in preparing a high quality manuscript, facilitate the review and assessment of revised manuscripts, and help to ensure that journal standards are maintained across the board. If your manuscript is accepted, the completed checklist will be published as supplemental material attached to the article online. Please download a copy of the checklist (pdf fillable form) at this link, for submission with your revised manuscript: https://tpc.msubmit.net/html/Author_Revisions_Checklist.pdf.

RESPONSE: The checklist has been followed and completed.

Supplemental materials should be restricted to large datasets and tables, presentation of replicates, and validation of reagents, methods, or genotypes. Any data that are used to support the major claims must be in the main manuscript. Supplemental figure legends must indicate what figure in the main manuscript is supported by the supplemental data presented. Please justify how each of the supplemental figures meet the criteria.

RESPONSE: We write in the legends of Supplemental Tables which figures they support in the main text.

Sampling methods and nature of "biological replicates" should be described precisely (i.e. different plants, parts of plants, pooled tissue, independent pools of tissue, sampled at different times, etc), along with a clear description of and rationale for any statistical analyses conducted. The reader should know exactly what was sampled; what forms the basis of the calculation of any means and statistical parameters reported. This is also necessary to ensure that proper statistical analysis was conducted.

RESPONSE: Sampling methods are described in detail.

<u>Reviewer #1:</u>

Taking advantage of barley (*Hordeum vulgare* L.) mutants at the *Viridis-k* locus and in vitro experiments, the present manuscript provides strong evidence that FdC2 is the natural electron donor for Mg- protoporphyrin IX monomethyl ester cyclase, XanL. This is a convincing manuscript with clear results and convincing conclusions. There is not much to criticize.

Other comments.

1. The data showed that VirK can drive the cyclase reaction with electrons provided by NADPH via FNR. Is light-driven electron transfer from PSI to FdC2 sufficient to drive the cyclase reaction? This could be assessed via an in vitro enzyme assay.

RESPONSE: This is indeed a very interesting experiment to do. We plan to do this with isolated PSI in the near future.

2. Did the authors, express the FdC2 Ala-118-Thr mutation recombinantly? Is the recombinant protein unstable, as suggested? How does Ala-118-Thr mutant FdC2 perform in the in vitro enzyme assay? Could it be that the Ala-118-Thr mutation impact the binding to XanL and therefore gets diminished in vivo?

RESPONSE: Again, this is an experiment we have in the pipeline. Presently, we do not know if the Ala-118- Thr mutation affects in vitro stability or binding to XanL

3. In the absence of XanL, FdC2 appears to be degraded, as suggested by the authors. What about mRNA expression, is the expression of *FdC2* affected due to the absence of XanL?

RESPONSE: We performed an RT-qPCR experiment in order to investigate the *HvFdC2* expression. (This experiment is the reason why the resubmission of this manuscripts took some time.) The results have been added and suggests that the effects are at protein level and not mRNA level.

4. Can FdC2 still rescue, when the transit peptide is removed?

RESPONSE: Again, an interesting question which has to be addressed in future experiments.

5. Another interesting question would be whether cyanobacterial ferredoxin (Fed2) could rescue the *Viridis-k* mutation in barley?

RESPONSE: It is highly relevant to test more Fds from barley and other organisms. In a previous study we demonstrated that spinach Fd obtained from Sigma can support the assay in vitro. We comment on this in the Discussion.

Reviewer #2:

The manuscript presents data indicating that a C-type ferredoxin is a required component for the Mg- PPIX-Me oxidative-cyclase involved in chlorophyll biosynthesis. It identifies the molecular basis of the barley *viridis-k* loci as being defective in the synthesis of this C-type ferredoxin, resulting in the *viridis-k* phenotype and presents both in vitro and in vivo evidence supporting these claims.

I enjoyed reading the manuscript as it was well written and the data support the claims.

The manuscript requires a small number of minor revisions as follows:

More details are required for the PAM imaging and cyclase assay methods.

1. It is unclear what type of PAM imaging is shown, I assume Fv/Fm with red being low or absent variable fluorescence and yellow to blue being an indication that functional PSII has formed. A scale would also be helpful on Fig. 5. Numerous modes of images and settings are possible with PAM imaging and no details or scale is provided in the figure legend. Details of the PAM are lacking in the methods such that this could not be reproduced.

RESPONSE: We have added the requested information concerning the PAM imaging.

2. The paper is about functional components of the oxidative cyclase and the methods should have enough details to repeat the cyclase assay results shown in Fig. 7 without referring to another publication.

RESPONSE: We added all details about our cyclase assay in the Material and Method section.

3. On line 565, The link to the NCBI SRA should be corrected (https://www.ncbi.nlm.nih.gov/sra) and the accession no. PRJNA686392 doesn't seem to exist. Please correct.

RESPONSE: Corrected.

Reviewer #3:

The *viridis-k* mutation has been around for a long time, from the von Wettstein collection, and it is gratifying to see that the gene has finally been identified, the mutations in the two alleles described, and a function ascribed to a unique ferredoxin. The authors present a complete analysis, including documentation of biochemical function (rather than just a generic pathway function).

The comments below are intended to improve accessibility of the work to a larger audience and to relate to other work on the cyclase enzyme. No additional experiments are necessary, just some time to re-write and re-word.

1. The writing needs to be tightened and made more accurate.

RESPONSE: Done to the best of our ability.

2. The authors define ferredoxin as electron acceptor of PS1 in abstract and elsewhere and then go on to say it does other things. Why not indicate that ferredoxins are FeS proteins that catalyze single electron transfer reactions. Then say that chloroplast ferredoxins are of the 2 Fe 2 S type, the most abundant of which is classic ferredoxin, encoded by the nuclear PETF locus, etc. Then cite perhaps the work of Guy Hanke and maybe Terauchi to say that plants and algae contain multiple ferredoxins whose pattern of expression (leaf vs root or induced by nitrate etc.) suggests that these other ferredoxins may play a role in other metabolic pathways, since ferredoxin is a central electron donor in cp metabolism.

This is also a problem in line 67 where authors write "Ferredoxin is the penultimate...". What is meant is that classic ferredoxin, product of PETF, is the electron carrier. Rather than saying "Alternatively, ferredoxin can..." they should say "This ferredoxin or other isoforms donate electrons for reduction reactions in sulfate and nitrate assimilation or for reduction of oxygen during oxidation of cp fatty acids". By the way acylACP desaturase from Shanklin is the prototypical di-iron enzyme that requires a ferredoxin.

RESPONSE: Thanks for the good suggestions. Rewritten as suggested.

3. I would suggest that figure 1 show the cyclase reaction and compare it in the legend to, for example, MMO a prototypical di-iron enzyme. MMO also requires a source of electrons (to reduce the second atom of O to water). Otherwise the typical plant biologist is not going to understand why an electron DONOR is needed when a 6 electron oxidation is mentioned in the introduction. The reader needs to understand that O2 is a substrate of the di-iron enzyme and that is what is doing the oxidation and a reductant is required to deal with the other atom. I do not know how exactly the ring V is created, but one can imagine - hydroxylation, oxidation to a ketone, to generate the adjacent acidic carbon which can deprotonate and then attach the porphyrin ring to give you ring V. I imagine it is essentially a concerted reaction?

RESPONSE: A new figure has been introduced as Figure 1. We explain the supposed reaction mechanism in the figure legend.

4. The loose writing makes some aspects confusing. For example, the abstract implies 3 ferredoxins but on page 3, the authors mention 4, and then 8 are mentioned in barley. See also lines 298-300. There is a supplemental figure that apparently shows the relationship of barley to Arabidopsis ferredoxins (characterized by Hanke). But a number of maize ferredoxins were also characterized by Hanke. All these relationships are quite close, yet chlorophyll biosynthesis has been conserved for a billion years, and presumably also the aerobic cyclase. I suggest that the supplemental figure (which I did not review) be moved to the main paper and that there be a meaningful discussion and naming of the ferredoxins, including with reference to the ones in green algae. Hanke or Carrillo might be able to review this aspect. I think this is a missed opportunity for the authors.

RESPONSE: An extended phylogenetic tree has been included in the main text, and a more stringent text has been applied to explain and view the different types of ferredoxins.

On line 100 on page 4, do the authors mean ortholog? This should be specified.

RESPONSE: We have changed to ortholog.

5. Ycf stands for hypothetical reading frame. It is unfortunate that even though a function in the cyclase reaction is now established, the name is still Ycf. The corresponding Chlamydomonas protein was called CGL78 (for conserved in the green lineage). In the GreenCut and GreenCut2, it is noted that enzymes unique to chlorophyll biosynthesis, such as Mg chelatase and the cyclase are conserved throughout the green lineage (Merchant et al. 2007, Karpowicz et al. 2011) and CGL78/Ycf54, plus CRD1/CHL27 and a couple of ferredoxins show up in that list. The Hsieh et al paper in MCP indicates co-expression of CGL78 and CHL27 (albeit not rigorously executed). These points should be made in the discussion. Perhaps in the context of the text on lines 255-257?

RESPONSE: Ycf54/CGL78/LCAA is required for the correct folding of XanL/CRD1/CHL27 and there is no evidence that Ycf54/CGL78/LCAA participates in the cyclase reaction. Therefore, Ycf54/CGL78/LCAA plays a marginal role in the present manuscript and we do not think it would support our conclusions concerning the function of VirK. We

are currently growing M2 plants of barley modified in the Ycf54/CGL78/LCAA gene by CRISPR. The naming will be addressed in that work following the nomenclature of the barley community for barley genes (Franckowiak and Lundqvist. 2010. Rules for nomenclature and gene symbolization in barley. Barley Genetics Newsletter 40:178-182.).

6. The authors should distinguish between protein names, gene names, and locus names. This locus has a name and the protein has a name, but the gene does not. The authors have the choice of using the locus name as a gene name (preferred) or coming up with a gene name. I would not recommend PETFx, since that can be confusing, nor do I recommend a name like FdC2, since that does not allow connection with say the Terauchi work where many ferredoxins were characterized. There are at least 9 ferredoxins in Chlamydomonas by the way.

RESPONSE: The proper name of the barley gene should be *Viridis-k* like the locus name. In order to follow the nomenclature of the barley community for barley genes (Franckowiak and Lundqvist. 2010. Rules for nomenclature and gene symbolization in barley. Barley Genetics Newsletter 40:178-182.), we therefore changed to *Viridis-k* (gene) and VirK (protein) in a more stringent way. When we previously used "HvFdC2", it was to help the reader to understand that we meant the barley ferredoxin ortholog of Arabidopsis FdC2.

7. It is interesting that a single point mutation can give such a strong phenotype. It would be interesting to express wt and mutant proteins for biophysical analyses. In the meantime, I wonder if the authors could present a structural model to show us where the mutation is on the structure? This may be something else that makes the paper interesting to a broader audience.

The few lines on the bottom of page 12 should be moved to the discussion.

RESPONSE: We are working on a biochemical characterization of the VirK protein. That work will include an analysis of the Ala-118-Thr mutation as well as a structural analysis. We moved the discussion-like text from the Result to the Discussion section as suggested.

8. There is no information on stability of the protein, only perhaps on its half-life, line 283.

RESPONSE: We have added a sentence in order to clarify that the mutation affects either the physical stability of the protein and/or makes it more prone to degradation by proteases.

There is some overlap between results and discussion. If the authors are asked to reduce the length of the manuscript by 10% or say discussion by 25% it would force them to avoid the repetition.

RESPONSE: Done to the best of our ability.

The source of antibodies (besides the ones that they made) is not indicated. Or at least I did not find it in the methods section. The immunoblot gives no technical information (Figure 6 legend). % gel, how many ug of protein, how long transferred, in what buffer, secondary antibody detection. Were all 4 proteins blotted in the same way? Ferredoxin is highly soluble whereas CHL27 is membrane associated. What was loaded? Were the antibodies made against barley proteins as implied in the legend or purchased from commercial sources?

RESPONSE: The source of the antibodies has now been clarified in the Material and Method section. Technical information has been added to the Figure legend.

There is no information on how the plants were grown including quality and quantity of light.

RESPONSE: This has now also been clarified in the Material and Method section.

<u>Reviewing Editor comments:</u>

The identification of the *VIRIDIS-K* locus, which encodes a ferredoxin with a C-terminal extension HvFdC2 that plays a dominant role in chlorophyll biosynthesis in barley, is a significant accomplishment. They also show that this is a functional Fd in vitro. Although this paper does not rule out complementary functions of one or more of the 7 other ferredoxins in the barley genome, it presents evidence that at least one of these may not functionally substitute for FdC2 using a novel transient complementation assay in vivo. The same research group recently showed that a canonical spinach Fd and FNR can support the activity of HvFdC2 in vitro (Stuart et al (2020) Aerobic barley Mg-

protoporphyrin IX monomethyl ester cyclase is powered by electrons from ferredoxin. Plants (Basel) 9). This raises the possibility that the negative result in the complementation assay with HvLFd1 shown in Fig. 5 may be due to the lack transient expression and/or proper localization. The authors might wish to comment on this.

RESPONSE: The chlorophyll deficient phenotype of the two *viridis-k* mutants is the strongest and best evidence that the VirK ferredoxin is the major electron donor to the cyclase reaction. The major point of the infiltration experiment is to demonstrate that the identified *Viridis-k/HvFdC2* gene is the correct gene that is deficient in the mutant. The mutant is called *viridis* because it can make a small amount of chlorophyll. Otherwise, it would be called *xantha*, which are the yellow chlorophyll devoid group of mutants. Both *viridis* and *xantha* mutants are lethal at the seedling stage. The small amount of chlorophyll made in the *viridis* mutants is most likely made with electrons from other ferredoxins. We have tried to clarify the importance of the fact that the *viridis-k* mutations are lethal.

TPC2021-RA-00018R1 2nd Editorial decision – *acceptance pending* **May 10, 2021**

We are pleased to inform you that your paper entitled "Barley Viridis-k links an evolutionary conserved C-type ferredoxin to chlorophyll biosynthesis" has been accepted for publication in The Plant Cell, pending a final minor editorial review by journal staff.

Final acceptance from Science Editor **May 21, 2021**
